# Spatial Patterns in the Distribution, Diversity and Abundance of Benthic Foraminifera around Moorea (Society Archipelago, French Polynesia)

**DOI:** 10.1371/journal.pone.0145752

**Published:** 2015-12-28

**Authors:** Olugbenga T. Fajemila, Martin R. Langer, Jere H. Lipps

**Affiliations:** 1 Steinmann-Institut für Geologie, Mineralogie und Paläontologie, Rheinische Friedrich-Wilhelms Universität, Bonn, Germany; 2 Department of Geological Sciences, Osun State University, Osogbo, Nigeria; 3 Department of Integrative Biology and Museum of Paleontology, University of California, Berkeley, California and J. D. Cooper Center, Santa Ana, California, United States of America; Ben Gurion University of the Negev, ISRAEL

## Abstract

Coral reefs are now subject to global threats and influences from numerous anthropogenic sources. Foraminifera, a group of unicellular shelled organisms, are excellent indicators of water quality and reef health. Thus we studied a set of samples taken in 1992 to provide a foraminiferal baseline for future studies of environmental change. Our study provides the first island-wide analysis of shallow benthic foraminifera from around Moorea (Society Archipelago). We analyzed the composition, species richness, patterns of distribution and abundance of unstained foraminiferal assemblages from bays, fringing reefs, nearshore and back- and fore-reef environments. A total of 380 taxa of foraminifera were recorded, a number that almost doubles previous species counts. Spatial patterns of foraminiferal assemblages are characterized by numerical abundances of individual taxa, cluster groups and gradients of species richness, as documented by cluster, Fisher *α*, ternary plot and Principal Component Analyses (PCA). The inner bay inlets are dominated by stress-tolerant, mostly thin-shelled taxa of *Bolivina*, *Bolivinella*, *Nonionoides*, *Elongobula*, and *Ammonia* preferring low-oxygen and/or nutrient-rich habitats influenced by coastal factors such as fresh-water runoff and overhanging mangroves. The larger symbiont-bearing foraminifera (*Borelis*, *Amphistegina*, *Heterostegina*, *Peneroplis*) generally live in the oligotrophic, well-lit back- and fore-reef environments. Amphisteginids and peneroplids were among the few taxa found in the bay environments, probably due to their preferences for phytal substrates and tolerance to moderate levels of eutrophication. The fringing reef environments along the outer bay are characterized by *Borelis schlumbergeri*, *Heterostegina depressa*, *Textularia* spp. and various miliolids which represent a hotspot of diversity within the complex reef-lagoon system of Moorea. The high foraminiferal Fisher *α* and species richness diversity in outer bay fringing reefs is consistent with the disturbance-mosaic (microhabitat heterogeneity) hypothesis.

Calculations of the FORAM Index (FI), a single metric index to assess reef vitality, indicate that all fore- and most back-reef environments support active carbonate accretion and provide habitat suitability for carbonate producers dependent on algal symbiosis. Lowest suitability values were recorded within the innermost bays, an area where natural and increasing anthropogenic influences continue to impact the reefs. The presence of habitat specific assemblages and numerical abundance values of individual taxa show that benthic foraminifera are excellent recorders of environmental perturbations and good indicators useful in modern and ancient ecological and environmental studies.

## Introduction

The Society Islands are located east of the tropical marine diversity hotspot with significance as recipients and redistributors of biotas via equatorial currents. They represent a transitional location between the high diversity assemblages of the coral triangle and the lower diversity biotas of the eastern Pacific. The beautiful coral rings and variety of habitats has made these islands ideal settings for coral health and reef management studies. While the coral community structure has been extensively studied [1–4], the foraminifera have received localized attention and are limited to case studies of specific environments [5–9] yet they are important members of tropical biotas, as monitoring aids for ecologic change, including global warming [10], and in understanding the history and development of islands. Foraminifera are prominent producers of calcium carbonate and contribute significantly to the calcium carbonate budget of coral reefs [11,12]. Given the present shortage of quantitative data on the spatial distribution of reef organisms, we studied an island-wide collection made in 1992 to analyze the structure and distribution of foraminiferal communities, to assess their diversity and to identify the dominant components in relation to their habitat. Moreover, coral reefs of Polynesia have experienced large-scale disturbances such as temperature increases, bleaching events [10], hurricanes and cyclones [13], human disturbance through sedimentation, pollution and damage on reefs [14,15], and outbreaks of *Acanthaster planci* which were followed by high rates of mortality [4,16–30]. Because of their abundance, ubiquity and rapid turnover rates, foraminifera are excellent indicators for studies of reef health and they preserve environmental information that is useful in interpreting changing ecological conditions and paleoecological studies. Our study, because of the distribution of collecting sites and number of species documented, provides a baseline for environmental changes since 1992. In light of this we provide new information on foraminiferal community structure and assess reef vitality using the FORAM Index (FI), a single metric index indicative of reef health and conditions for carbonate accretion [31,32].

The study of foraminifera in the French Polynesian Islands dates back to the H.M.S. *Challenger* Expedition (1873–1876) when 10 new species were described and documented from around the Society archipelago [33]. Later the Albatross expedition of 1899–1900 provided new material from the Tuamotu, the Marquesas and the Society Islands [34–37]. However, these efforts were followed by longer years of inattention in the Polynesian corridor until in the 1970s and 80s. Le Calvez and Salvat [38] and Vénec-Peyré and Salvat [39] gave concise reports on the foraminiferal assemblages from the reef-lagoon system of the island of Moorea and the Scilly Atoll (French Polynesia). Salvat and Vénec-Peyré [40] recognized 25 living foraminifera and concluded that the majority of the species are cosmopolitan and the population is affected by dwarfism. By 1985, Vénec-Peyré listed a total of 182 species that belonged to 39 families [5,41]. To date, this published list of species represented the largest source of information to assess the diversity of foraminiferal communities from Polynesia. In light of recent large-scale surveys on foraminifera from other areas of the Indo-Pacific where up to 1000 species were recorded [42–45], the number currently known appears to be comparatively low. Vénec-Peyré [6,7,46] also noted that lagoonal biocoenoses are less diverse compared to assemblages from the outer slope and showed that substrate types control the composition of assemblages. Later, she examined living foraminifera on both sides of the barrier reef across a section along the northwestern part of Moorea. A total of 87 species were recorded, with 62 in the back-reef area (fringing reef, channel and barrier reef) and 72 on the outer slope; 47 were common to both zones. Mangrove foraminifera were the focus of research by Langer and Lipps [9] with a total of 96 species recorded from introduced mangrove habitats, showing that the assemblages are distinct from other nearshore habitats.

The present paper reports the results of the first island-wide study of foraminifera around Moorea. This study presents new information on the structure, patterns of distribution and diversity of benthic foraminiferal assemblages with respect to their role as environmental indicators. It also contributes to the worldwide biogeographic studies of larger benthic foraminifera, which would form an integral part of the diversity gradients from the epicenter and hotspot of the Coral Triangle [47,48] towards the flanks of the eastern Pacific and into the Indian Ocean.

## Materials and Methods

### Study area and sampling sites

**Fig 1:**
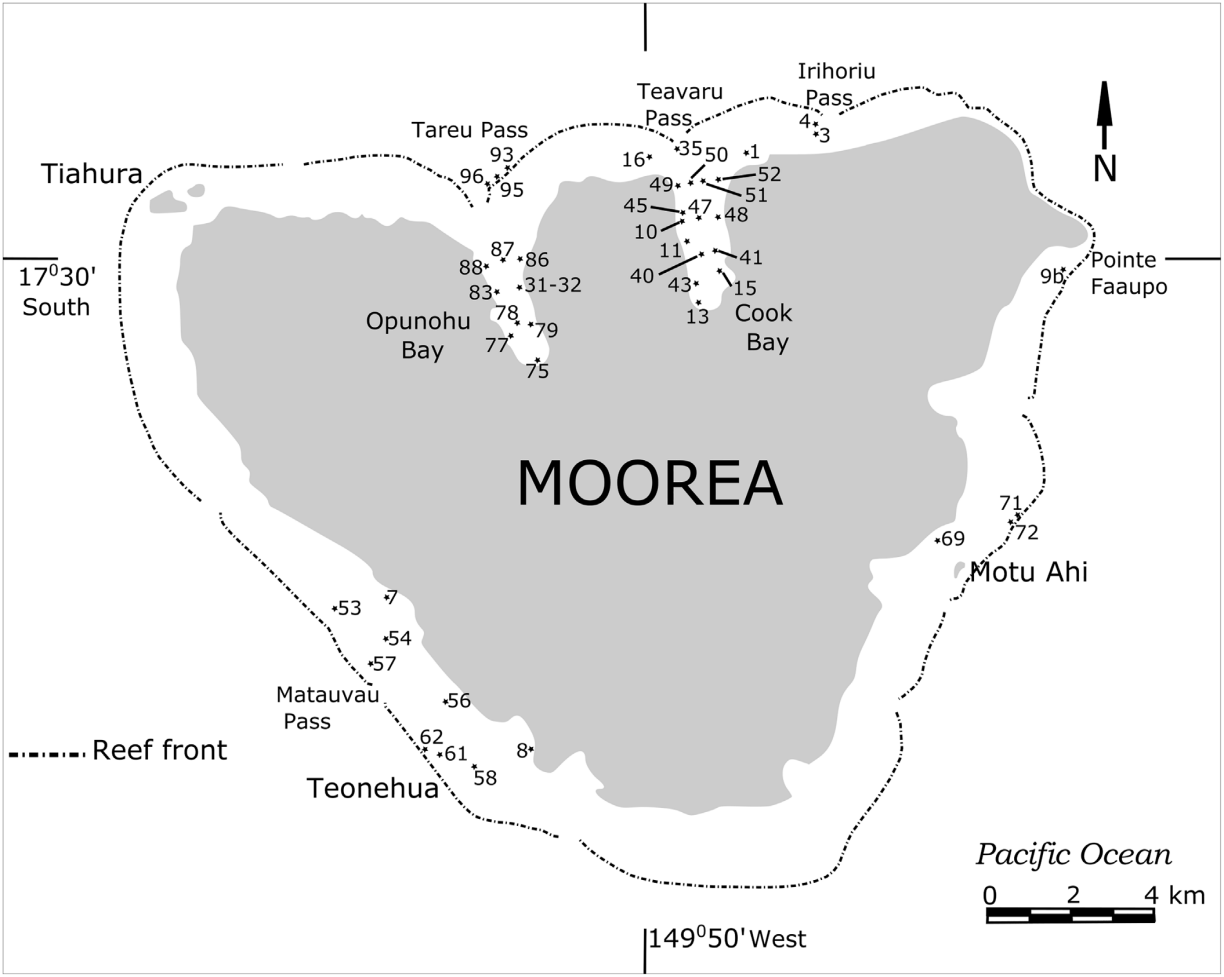
Location of the 45 sample station around Moorea, French Polynesia (for details see Table 1).

This study was conducted around the high island of Moorea, French Polynesia (17°30S, 149°50W), just 25 km NW of Tahiti and is part of the Society Archipelago in the South Pacific. The island, of volcanic origin, is 1.2 million years old and is surrounded by an encircling barrier reef only a few thousand years old, at least at their present sea level elevation. Water exchange from within the barrier and the open ocean is controlled by several larger and smaller passes in the barrier reef. The barrier reef encloses a shallow back-reef and lagoonal area that ranges between 500 and 1000m in width. On the northern side are two deep bays (Opunohu and Cook’s Bays) that give the island a “heart-shaped” appearance. The island has a total area of 134 km^2^, a circumference of 61 km, a height of 1207 m, and has 49 km^2^ of reefs, lagoons and nearshore habitats (Fig 1). Forty-five sample stations were selected around the island within the bays, lagoons, and back- and fore-reef environments for good representation of environmental habitats. These comprise the shallow water habitats of Opunohu Bay and Tareu Pass, Cook’s Bay and Teavaru Pass, Irihoriu Pass, Teonehua and Matauvau and near Motu Ahi and Point Faaupo (Fig 1). Samples were collected in 1992 from the sediment surface by filling plastic bags (20x40 cm) with substrata from the top 2cm while Scuba diving and snorkeling. The sampling sites cover a depths range from 0-40m. All samples were washed over 63μm mesh sieves and dried at 50°C in an oven overnight. Foraminifera were picked from each sample and individuals of each species were counted. Live foraminifera were grouped with dead tests in our study because our aim was to provide general environmental and biogeographic data useful in paleoecology. Our samples are thus time-averaged and as such provide an effective means of defining reefal habitats [44].

More than 16,000 individuals were picked, identified to species level and counted wherever possible. Based on their ecological roles in warm-water environments, all genera were categorized into three functional groups (symbiont-bearing, heterotrophic and stress-tolerant opportunistic taxa; [31]) and percent abundances of each group were calculated. Images of representative species were taken by Scanning Electron Microscope (SEM) and arranged into the plate using the Adobe Photoshop CS6. All samples and illustrated specimens will be deposited in the micropaleontological collections of the Museum of Paleontology, University of California, Berkeley (no. MF9218 to MF9299 and specimen nos. 16310 to 16399). This work was done under permit No. 568/BCO from Haut-Commissariat de La Republique en Polynesie Francaise.

The reefal, bay and lagoonal habitats exhibit specific environmental features: these include the nature and composition of sediments, fresh water runoff and the coverage by mangroves, algae and corals. The shoreline habitats are distributed along the shallow lagoon. Within the innermost bays and at several shore sites mangroves (*Rhizopora stylosa*) trees introduced in 1937 line the coastal areas. These habitats are mostly muds and roots in mangrove forests, in shore sands and organic-rich mud/silt under overhanging native *Hibiscus tiliaceus*,in *Paspalum vaginatum* salt grass marshes, and in some sand and beach rock.

Fringing reefs are located along the shores of the outer bays and are also present as patches along the surrounding coast. Their depth usually does not exceed 3 meters. The coral assemblage is characterized by *Synarea*, *Psammocora*, *Montipora* and *Acropora*. Algal vegetation within the bay is dominated by *Halimeda*, *Padina*, *Microdictyon*, *Caulerpa*, *Turbinaria* and *Porolithon*. The composition of sediments towards the outer bay and the coastal fringing reefs varies in general from site to site by increases from muddy and silty sediments to sandy and coarse-grained carbonaceous sand and rubble along the coast.

The reef barrier is up to 400 meters wide, formed by a shallow reef flat (2m) and bordered by a reef crest. The outer reef slope comprises furrowed platforms, buttresses and valleys and downward sloping platforms to very deep waters. *Acropora*, *Montipora*, and *Pocillopora* corals are among the dominant genera in the fore-reef areas. Sediments comprise coral rubble, and coarse detrital sand that accumulates in furrows and valley bottoms. The bottom then drops steeply to the ocean floor (2000m).

Tides at Moorea are semi-diurnal with a maximum range of <0.4 m. Current velocities are generally low (0.5 km/h) but may reach up to 3.5 km/h in the channels intersecting the passes [8,49]. Lagoon water temperature is around 27°C with variations of up to 5°C. Waters that cross the reef barrier into the lagoon flows back through the passes and channels into the open ocean. The average residence time for waters in this system has been estimated to be around 6 hours [50]. Being located in the middle of an ocean and far from continental runoff, the waters surrounding Moorea are oligotrophic, except within the innermost bays where occasional runoff from the island and sewage disposal eutrophicate Moorea’s pristine waters. Rarely, storms push seawater into the lagoon sufficient to flood the mangrove, marshes, and other near shore habitats and human infrastructure. These conditions do not last for more than a few days but may serve to distribute organisms to new locations within the lagoon.

To determine the structure in the foraminiferal data set we performed Q-mode clustering techniques with the paired group algorithm using the Bray-Curtis dissimilarity. Cluster analysis is a large-scale analytical procedure to detect structural entities within complex data sets. This entails data mining and patterns discovery. For the cluster analysis, the data was imported into PAST software and analyzed [51]. This technique grouped together samples with similar faunal assemblages and revealed a typology of environmental signatures embedded in a hierarchical dendrogram of foraminiferal assemblages. The full set of samples was subjected to Q-mode cluster analyses and the resulting dendrogram referenced to collection points.

For structural refinement a principal component analysis (PCA) was conducted to identify similarities and differences among foraminiferal assemblages. This is helpful in a multivariate analysis to structure and visualize larger data sets by reducing a large number of variables to a few linear combinations (principal components). The eigenvectors are mutually perpendicular axes defining the coordinate system of the space and the eigenvalues give a measure of the ‘importance’ of each new axis to the data [52]. For PCA the most abundant genera were selected for this analysis (see Table 2). They include: *Ammonia*, *Amphistegina*, *Bolivina*, *Elphidium*, *Hauerina*, *Homotrema*, *Miliolinella*, *Peneroplis*, *Quinqueloculina*, *Schlumbergerina*, *Sorites*, *Textularia* and *Triloculina*. The data sets were entered into PAST software and analyzed. To measure diversity, species richness was determined for each sample and illustrated by the Fisher *α* diversity index (Table 1; [53,54]). For this, the total number of individuals was plotted against the total number of species using the PAST software. This allows a comparison of foraminiferal assemblage diversity (species richness) with regards to the sampled habitats. Ternary diagrams were employed to accentuate assemblage differences among reefal habitats, by plotting percent abundances of wall structural types present in each sample [54,55].

**Table 1:** Sample sites showing location, depth, calculated Fisher α indices, FORAM—Index (FI) values, and total number of genera and species recorded in individual environments.

Sample no	Location		Depth (m)	Fisher α indices	FORAM—Index	No of Genera	No of Species	No of Specimen
Lagoon	Longitude	Latitude						
M1	17° 28’ 53.96” S	149° 48’ 55.67” W	8–10	14.97	3.4	18	53	503
M3	17° 28’ 36.49” S	149° 47’ 59.91” W	1.5	16.03	2.9	16	51	377
M4	17° 28’ 31.02” S	149° 48’ 00.02” W	1	3.385	3.2	12	14	280
M51	17° 29’ 01.85” S	149° 49’ 16.53” W	20	2.512	1.7	13	13	449
**Back-reef**								
M16	17° 28’ 57.96” S	149° 49’ 54.73” W	3	14.04	3.6	14	47	396
M35	17° 28’ 58.07” S	149° 49’ 26.67” W	2.5	30.85	2.5	47	75	325
M53	17° 33’ 24.12” S	149° 53’ 11.43” W	1–1.4	16.74	3.0	34	50	321
M54	17° 33’ 47.14” S	149° 52’ 41.24” W	1.4	16.73	3.0	35	51	338
M58	17° 35’ 15.52” S	149° 51’ 42.40” W	1.4	8.227	3.8	24	31	369
**Fore-reef**								
M9b	17° 30’ 10.14” S	149° 45’ 40.19” W	20	1.59	6.4	7	8	242
M61	17° 35’ 10.39” S	149° 52’ 01.11” W	20–25	4.2	7.7	13	19	383
M62	17° 35’ 04.42” S	149° 52’ 11.39” W	20–25	12.84	4.9	29	43	353
M71	17° 32’ 25.53” S	149° 45’ 50.31” W	20	5.133	6.6	17	22	368
M72	17° 32’ 33.89” S	149° 45’ 59.66” W	20	10.26	6.9	30	38	410
M93	17° 28’ 49.43” S	149° 51’ 08.23” W	12	10.87	5.7	30	40	422
M95	17° 29’ 03.78” S	149° 51’ 26.33” W	22	8.99	6.5	26	36	492
M96	17° 29’ 05.72” S	149° 51’ 28.35” W	20	16.44	5.4	32	52	310
**Mangrove**								
M7	17° 33’ 21.26” S	149° 52’ 39.47” W	0–0.5	6.914	2.1	9	30	526
M8	17° 33’ 02.70” S	149° 51’ 08.71” W	0–0.5	3.856	1.8	11	17	318
**Bay inlets**								
M13	17° 30’ 21.29” S	149° 49’ 19.95” W	1.5	15.39	1.6	35	48	329
M15	17° 30’ 04.87” S	149° 49’ 04.03” W	3	13.32	1.7	34	48	520
M40	17° 29’ 59.57” S	149° 49’ 15.90” W	10	5.298	1.3	16	40	503
M41	17° 29’ 57.90” S	149° 49’ 08.45” W	10–15	6.476	1.3	17	25	304
M43	17° 30’ 15.68” S	149° 49’ 23.40” W	6	6.682	1.4	18	25	275
M47	17° 29’ 26.54” S	149° 49’ 17.87” W	15–20	14.07	1.7	34	46	370
M50	17° 29’ 10.35” S	149° 49’ 26.16” W	20–25	17.5	1.8	34	51	320
M56	17° 34’ 19.16” S	149° 52’ 01.39” W	3.6	14.79	2.0	27	47	350
M75	17° 30’ 01.20” S	149° 51’ 04.32” W	1.4	5.412	1.5	22	22	315
M77	17° 30’ 42.00” S	149° 51’ 20.43” W	20	19.53	1.9	38	57	341
M78	17° 30’ 33.77” S	149° 51’ 17.32” W	24.5	15.62	1.6	24	47	300
M79	17° 30’ 34.84” S	149° 51’ 07.45” W	20	10.78	1.7	25	37	321
M83	17° 30’ 22.85” S	149° 51’ 27.00” W	14	21.71	2.6	41	59	311
**Fringing reefs**								
M10	17° 29’ 43.24” S	149° 49’ 28.84” W	0.5	17.55	1.9	36	52	327
M11	17° 29’ 55.20” S	149° 49’ 24.34” W	2–2.5	3.235	2.4	35	53	353
M31	17° 30’ 15.69” S	149° 51’ 12.83” W	1.4	31.19	2.9	41	77	358
M32	17° 30’ 15.69” S	149° 51’ 12.83” W	1.4	17.51	2.6	41	56	443
M45	17° 29’ 29.40” S	149° 49’ 31.02” W	0.5–2.5	22.48	2.4	51	62	273
M48	17° 29’ 27.46” S	149° 49’ 08.07” W	0.5–2.5	30.52	2.1	46	71	292
M49	17° 29’ 11.57” S	149° 49’ 39.62” W	8–10	25.9	2.4	49	69	364
M52	17° 29’ 09.77” S	149° 49’ 06.10” W	10–15	23.94	2.7	37	57	238
M57	17° 34’ 01.56” S	149° 52’ 49.55” W	1.3	15.5	2.3	32	46	295
M69	17° 32’ 47.19” S	149° 46’ 42.45” W	2.5	19.52	2.5	31	54	302
M86	17° 29’ 51.61” S	149° 51’ 16.16” W	0.5–2.5	26.79	3.5	46	72	373
M87	17° 29’ 52.23” S	149° 51’ 28.67” W	38	27.5	2.3	48	70	340
M88	17° 29’ 55.90” S	149° 51’ 43.21” W	0.5–2.5	23.1	4.2	50	70	466

**Table 2:** Generic categorization of functional groups of foraminifera based on ecological preferences in warm water environments [31,64,65].

Symbiont-bearing	*Amphistegina*, *Amphisorus*, *Assilina*, *Borelis*, *Coscinospira*, *Heterostegina*, *Monalysidium*, *Parasorites*, *Peneroplis*, *Sorites*.
Opportunistic	*Ammonia*, *Bolivina*, *Bolivinella*, *Bulimina*, *Buliminella*, *Elongobula*, *Elphidium*, *Fursenkoina*, *Hopkinsina*, *Loxostomina*, *Nonionoides*, *Reusella*, *Sigmavirgulina*, *Trifarina*.
Heterotrophic	*Abditodentrix*, *Acervulina*, *Acupeina*, *Adelosina*, *Agglutinella*, *Ammobaculites*, *Ammoscalaria*, *Anomalinella*, *Articulina*, *Baggina*, *Brönnimannia*, *Cancris*, *Caronia*, *Cerebrina*, *Cibicides*, *Cibrobaggina*, *Clavulina*, *Conicospirrillinoides*, *Cornuspira*, *Cyclammina*, *Cycloforina*, *Cymbaloporetta*, *Discorbinella*, *Dyocibicides*, *Edentostomina*, *Eponides*, *Euthymonacha*, *Falsagglutinella*, *Fijiella*, *Fisherinella*, *Fissurina*, *Haddonia*, *Haynesina*, *Hauerina*, *Homotrema*, *Inaequalina*, *Lagena*, *Lobatula*, *Massilina*, *Mesosigmoilina*, *Miliola*, *Miliolinella*, *Milletiana*, *Mimosina*, *Murrayinella*, *Neoconorbina*, *Nubeculina*, *Nodophtalmidium*, *Oolina*, *Palliotella*, *Paratrochammina*, *Pitella*, *Planispirillina*, *Planispirinella*, *Planogypsina*, *Porosononion*, *Procerolagena*, *Pseudogaudryina*, *Pseudohauerina*, *Pseudohauerinella*, *Pseudomassilina*, *Pseudononion*, *Pseudoschlumbergerina*, *Pseudotriloculina*, *Pyrgo*, *Quinqueloculina*, *Reophax*, *Rhabdammina*, *Rosalina*, *Rotorbis*, *Sagrinella*, *Sagrinopsis*, *Sahulia*, *Schlumbergerina*, *Septotextularia*, *Sigmoihauerina*, *Sigmoilinita*, *Sigmoilopsis*, *Siphonaperta*, *Siphogenerina*, *Siphonina*, *Siphotrochammina*, *Sphaerogypsina*, *Spirillina*, *Spiroloculina*, *Spirophthalmidium*, *Spirosigmoilina*, *Stictogongylus*, *Textularia*, *Tretomphalus*, *Triloculina*, *Trimosina*, *Trochammina*, *Valvulineria*, *Verneuilina*, *Vertebralina*, *Wiesnernella*.

For supraspecific identification we follow Loeblich and Tappan [56] and modifications proposed by Hottinger et al. [57]. For species identifications we have applied the concepts of the nearest complete faunal studies from the Sahul Shelf [43], off Malaysia west of New Guinea [58], the Great Barrier Reef [59], Madang Lagoon and Chuuk Atoll [44,60], New Caledonia [42,61] and Ningaloo Reef [45].

To assess the general state of reefal conditions, the FORAM-Index (FI) was calculated at each sample station [30,31,62]. The FI is a single metric index to determine the impact of environmental stressors on coral reef environments and to assess whether the quality of water is sufficient to support mixotrophy (algal symbiosis). This measure is based on foraminiferal shells present in the sediment and is independent of coral populations. By virtue of foraminiferal abundance, the index allows a rapid and cost-effective assessment of environmental conditions. Calculation of FI depends on the relative abundances of symbiont-bearing, opportunistic and heterotrophic taxa and is particularly meaningful in populations that “integrate anthropogenic and natural stressors on the organisms over time” [63]. The FORAM Index is calculated using the following equation:

$$\text{FI = (10 x }P_{s}\text{) + }P_{o}\text{ + (2 x }P_{h}\text{)}$$

where FI = FORAM Index, Ps = Number of Larger Symbiont-bearing species/T, Po = Proportion of the opportunistic taxa/T, P_h_ = Proportion of smaller heterotrophic taxa/T, and T = the total number of foraminifera counted (for details see [31]).

## Results

### Structure of foraminiferal assemblages

**Fig 2:**
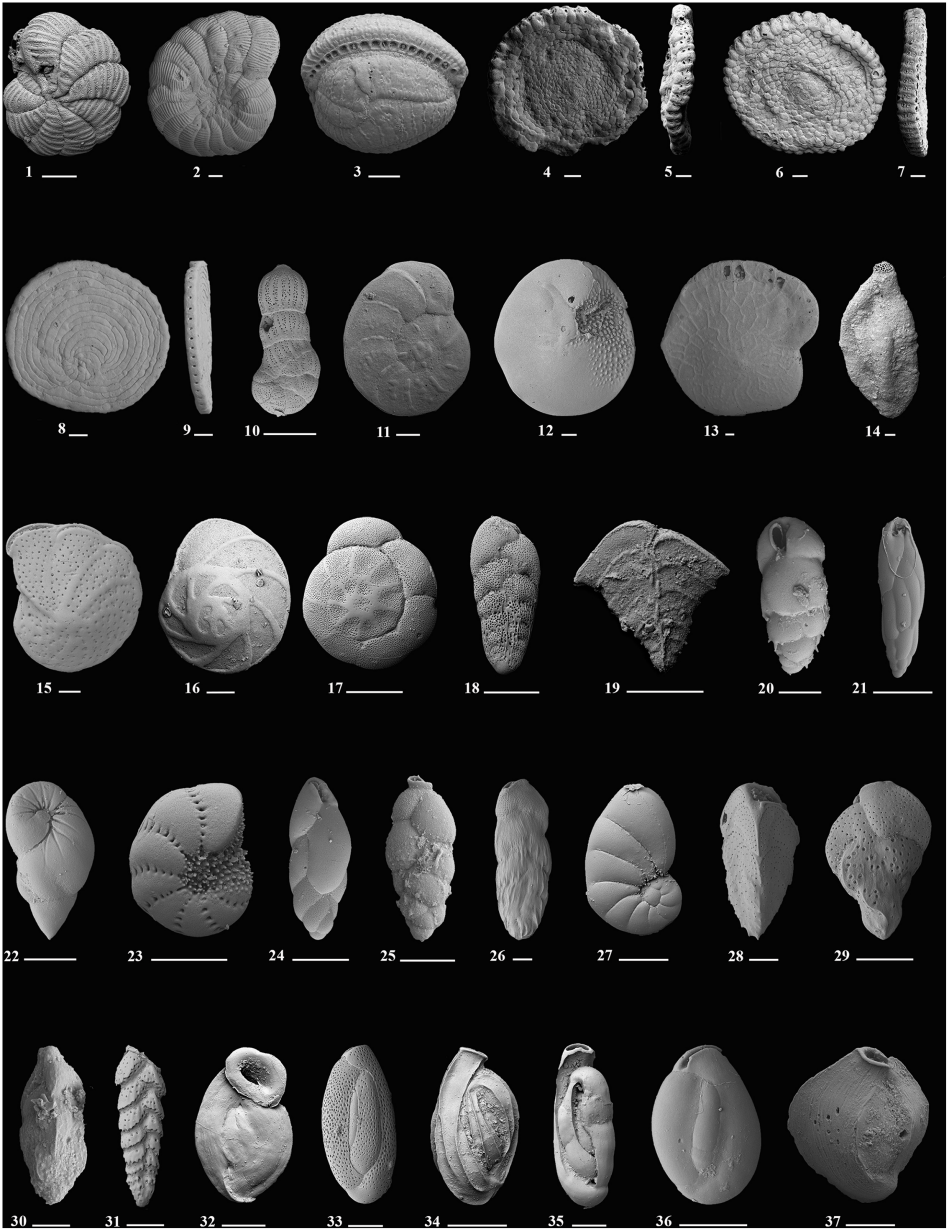
Scanning electron micrographs of selected species of indicator foraminifera characterizing the major cluster habitats around Moorea Island. **Species that bear symbionts**: **1**. *Coscinospira hemprichii* Ehrenberg; **2**. *Peneroplis pertusus* Forskål; **3**. *Borelis schlumbergeri* Reichel; **4**, **5**. *Amphisorus hemprichii* Ehrenberg (Scale bar is 200μm); **6**, **7**. *Sorites orbiculus* Ehrenberg; **8**, **9**. *Parasorites orbitolitoides* Hofker; **10**. *Monalysidium confusa* McCulloch; **11**. *Assilina (Operculina) ammonoides* Schröter; **12**. *Amphistegina lessonii* d’Orbigny; **13**. *Heterostegina depressa*, d’Orbigny; **Species associated with the symbiont-bearing taxa**: **14**. *Schlumbergerina alveoliniformis* Brady; **15**. *Anomalinella rostrata* Brady; **16**. *Eponides repandus* Fitchel and Moll; **Opportunistic species**: **17**. *Ammonia tepida* Cushman; **18**. *Bolivina striatula* Cushman **19**. *Bolivinella elegans* Parr; **20**. *Bulimina* sp. 1 (Scale bar is 50μm); **21**. *Buliminella elegantissima* d’Orbigny; **22**. *Elongobula spicata* Cushman and Parker; **23**. *Elphidium oceanicum* Cushman; **24**. *Fursenkoina schreibersiana* Czjzek; **25**. *Hopkinsina pacifica*, Cushman; **26**. *Loxostomina limbata*, Brady; **27**. *Nonionoides grateloupi* d’Orbigny; **28**. *Reusella pacifica* Cushman and McCulloch; **29**. *Sigmavirgulina tortuosa* Brady; **30**. *Trifarina bradyi* Cushman; **Heterotrophic species**: **31**. *Sagrinella convallaria*, Millett; **32**. *Wiesnerella auriculata* Egger; **33**. *Quinqueloculina cf*. *Q*. *semireticulosa* Cushman; **34**. *Quinqueloculina funafutiensis*, Chapman; **35**. *Quinqueloculina exsculpta* Heron-Allen and Earland; **36**. *Quinqueloculina eburnea* d’Orbigny; **37**. *Quinqueloculina cuvieriana* d’Orbigny. Scale bar is 100μm for all magnifications.

A total of more than 16,000 benthic foraminifera belonging to 380 species including agglutinated, perforate-hyaline and imperforate-porcellaneous types were recovered from the 45 samples from around Moorea island. The number of species increases to 422 when additional species listed in Vénec-Peyré [41] and Langer and Lipps [9] are included (see S1 List). The foraminiferal assemblages represent 127 genera. Agglutinated foraminifera account for 16 genera, while porcelaneous and hyaline perforate have 45 and 66 respectively. Ten symbiont-bearing foraminiferal genera include *Amphistegina*, *Amphisorus*, *Assilina (Operculina)*, *Borelis*, *Coscinospira*, *Heterostegina*, *Monalysidium*, *Parasorites*, *Peneroplis* and *Sorites* (Table 2 and Fig 2). All species were categorized into symbiont-bearing, heterotrophic and opportunistic taxa (Table 2). Symbiont-bearing species represent 18% of all individuals counted, heterotrophic taxa make up 66% and opportunistic species account for 16% of all specimens.

Symbiont-bearing individuals are particularly abundant at some reefal sites (M9b, M61-62, M71-72, M93, M95-96) where they represent up to 72% of the total foraminifera. The number of individuals in this group gradually increases from back-reef settings to fore-reef habitats. They constitute between 0–20% of the samples in Cook’s Bay, 0–28% in Opunohu Bay, 7–69% at Motu Ahi and 1–72% at Teonehua. *Amphistegina lessonii* is the most abundant species in this group. It is recorded with particularly high numbers within the reefal settings.

**Fig 3:**
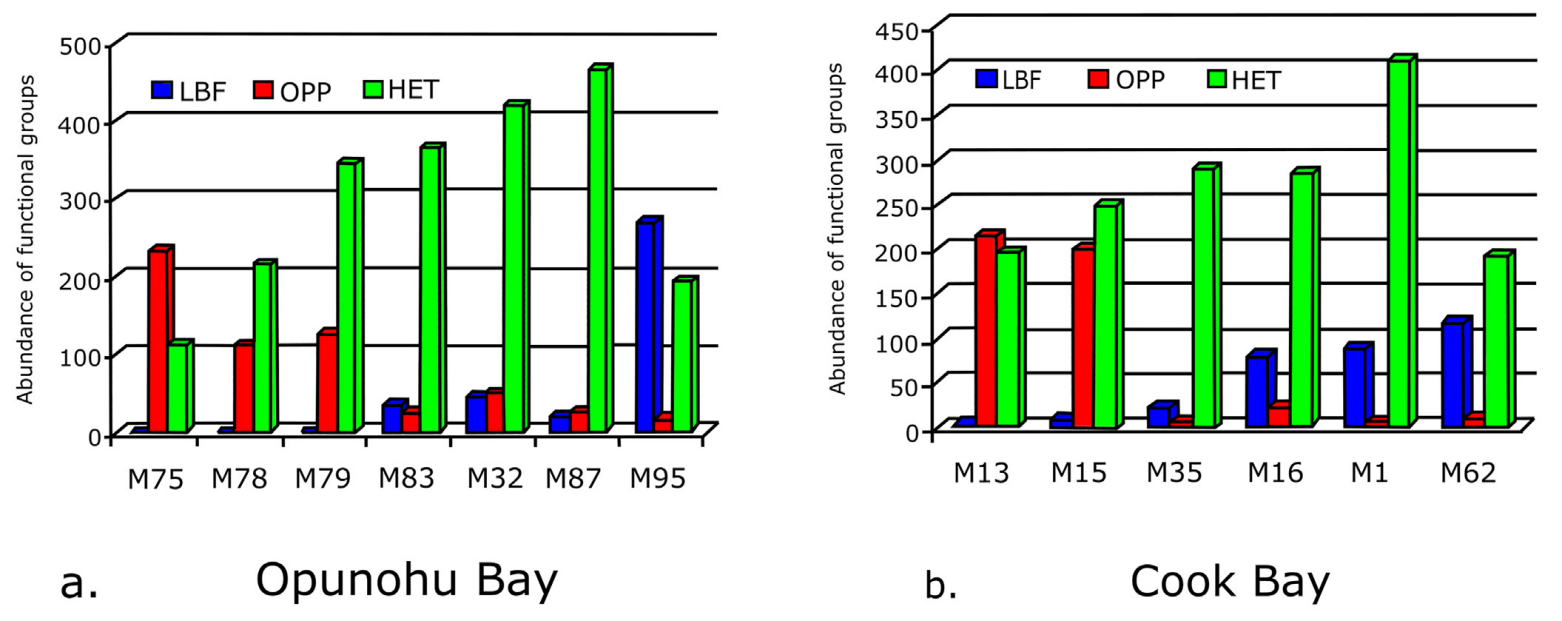
Abundance records of larger symbiont-bearing (LBF), Opportunistic (OPP) and Heterotrophic (HET) foraminifera within a) Opunohu and b) Cook’s Bay.

Opportunistic taxa (*Ammonia*, *Bolivina*, *Bolivinella*, *Elphidium*, *Elongobula*, *Hopkinsina*, *Loxostomina* and *Nonionoides*) are quite abundant in the bay inlets of Opunohu and Cook’s Bay. Other typical opportunistic species include *Bulimina*, *Buliminella*, *Fursenkoina*, *Reusella*, *Sigmavirgulina* and *Trifarina* occur rarely. Percent abundances of all opportunistic taxa range between 1–67% in Cook’s and Opunohu Bays, 7–69% at Motu Ahi and 0–32% at Teonehua. In general, they are more abundant in the lagoon and bays (Fig 3a). Their frequency decreases towards the back and fore-reef sites where symbiont-bearing taxa often dominate the assemblages.

Heterotrophic species of foraminifera occur in all samples and form the bulk of the overall number of species recorded. They range between 25 and 90% of foraminifera in all the samples. Highest abundances were recorded at various lagoon, bay and fringing reef sites (M3, M4, M7, M13, M16, M35, M51, M79, M83, M86) where they represent between 47and 90 percent of all individuals counted (Fig 3b).

### Fisher α diversity indices

**Fig 4:**
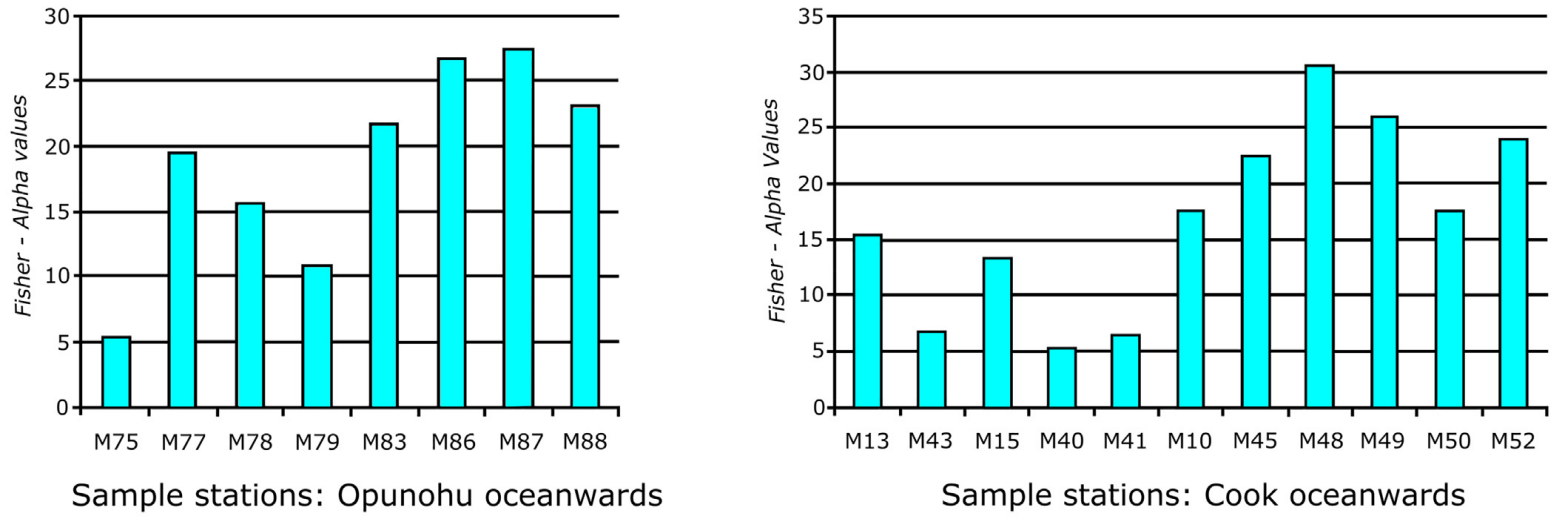
Increase in *Fisher α* diversity indices from the bays to the back-reef habitats.

Assemblage indices revealed more genera in the fringing-reef than in the fore-reef, bay and lagoonal environments (Table 1). In general, the Fisher *α* diversity indices increase from the bays towards the lagoonal sites, towards fringing reefs, and the back- and fore-reef sites. This trend is particularly evident in Opunohu and Cook’s Bay, where species richness and Fisher *α* diversity rises along transects from bay inlets towards fore-reefs (Fig 4). Individual assemblages, however, display a substantial species richness range. Within the bay inlets, especially in the Opunohu Bay, the Fisher *α* index range from 5 (innermost bay sample sites) to 27 at the outer most oceanward sample station. A similar trend was recorded at Cook’s Bay, where Fisher *α* diversity increases from 6 to 30 towards the open ocean.

### Cluster Analysis

**Fig 5:**
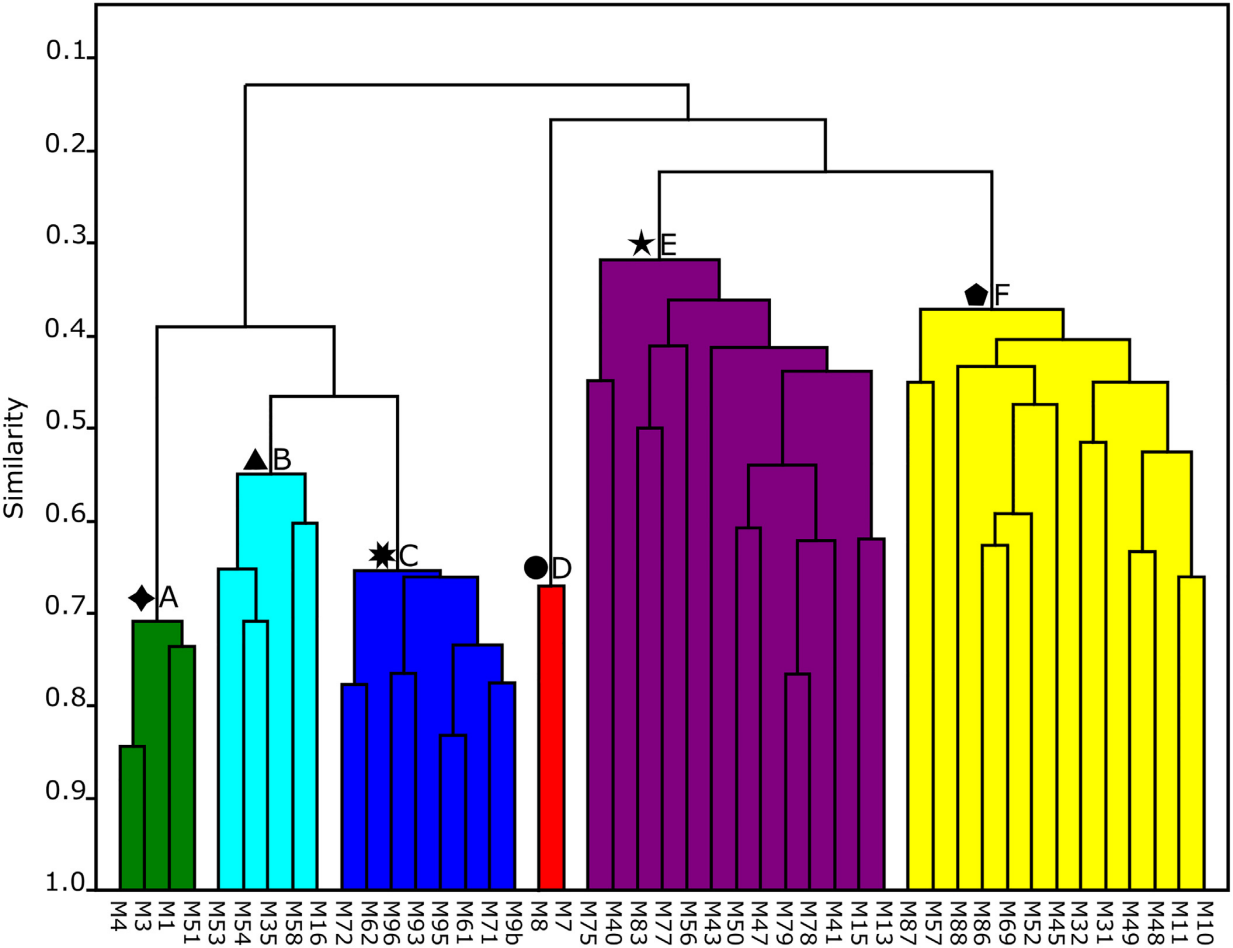
Q-mode cluster diagram of sample sites exhibiting the presence of 6 major cluster groups.

**Fig 6:**
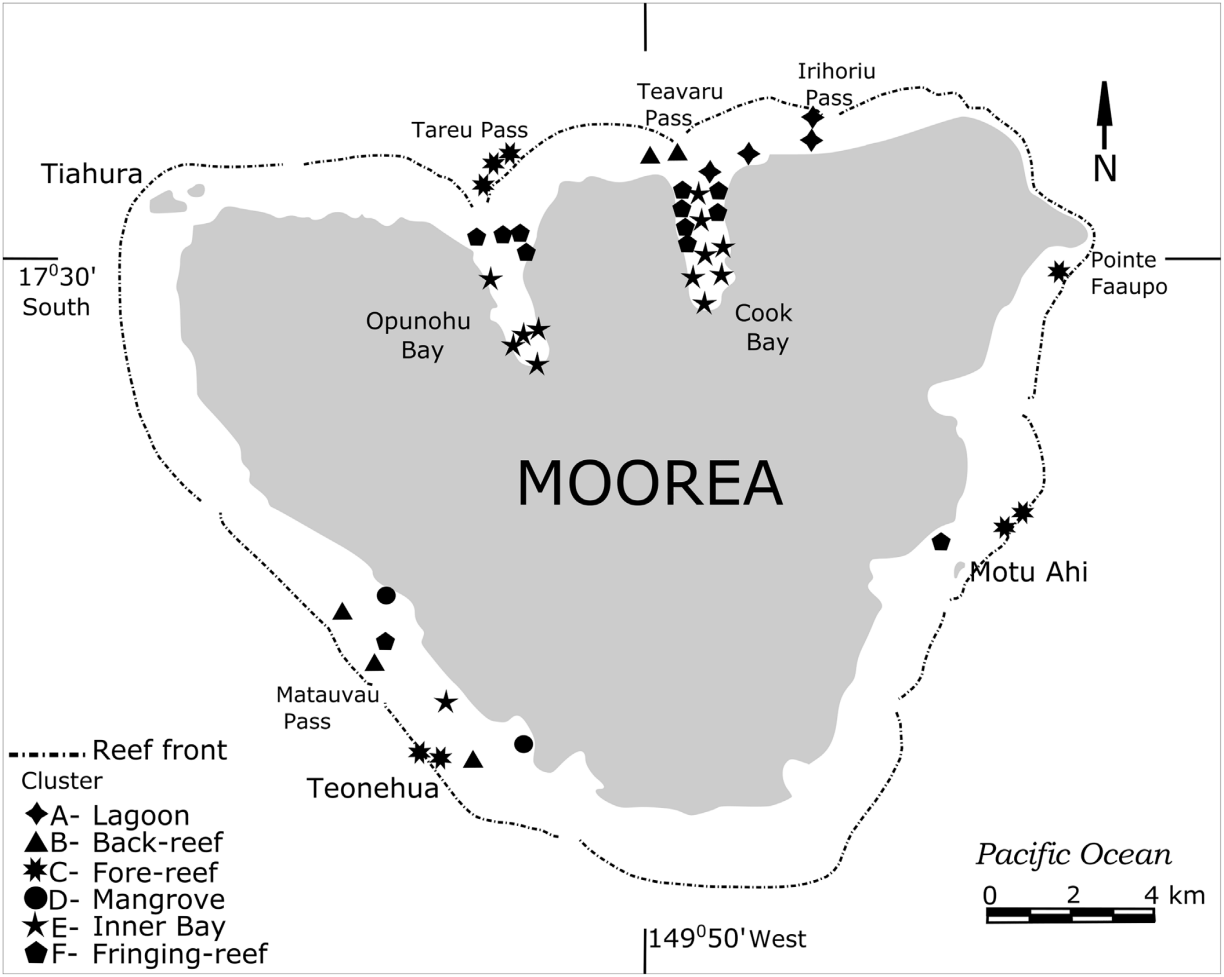
Map showing locations of the clusters stations. The symbols represent the clusters defined in Fig 5.

Cluster analysis comparing the composition and abundance data of foraminifera in all sample stations revealed the presence of six clusters separated in two major groups (cluster A-F, Fig 5). Sample sites belonging to individual clusters were marked with symbols and are plotted in Fig 6. The figure shows that individual clusters characterize specific habitats and environmental conditions. These include bays, fringing reefs, lagoon, coastal mangrove areas, and back- and fore-reefs.

#### Cluster A (Lagoon)

Cluster A (Lagoon) comprises samples from the northwestern part of the island along the Irihoriu Pass and environments of the lagoon floor with coral rubble, fine calcareous sand and limited algal cover. It is characterized by smaller miliolids, some larger symbiont-bearing foraminifera and *Homotrema rubra*, a permanently attached taxon. This cluster includes at least 100 species of foraminifera among which *Homotrema rubra*, *Amphistegina lessonii*, *Bolivina striatula* and *Peneroplis pertusus* are the most abundant species. Other species like *Hauerina pacifica* and *Sorites orbiculus* occur in minimal proportions.

#### Cluster B (Back-reef)

Cluster B is associated with back-reef slopes and contain sample sites from the Teavaru Pass at Cook’s Bay, and from Teonehua and Matauvau in the southwest. The cluster contains a diverse assemblage of 135 species of foraminifera including thick-shelled miliolids and various symbiont-bearing taxa (*Ampistegina* spp., *Sorites orbiculus*, *Peneroplis* spp.). The back-reef assemblages are characterized by frequent occurrences of *Homotrema rubra*, *Amphistegina lessonii*, *Millettiana millettii*, *Peneroplis pertusus*, *Miliolinella oceanica*, *Quinqueloculina seminula* and *Q*. *poeyana*. Species of *Elphidium*, *Bolivina* and *Ammonia* occur in minimal quantities.

#### Cluster C (Fore-reef)

Cluster C includes all sites from fore-reef to 30m depth and reef-top habitats. A total of eight samples belong to this cluster: three from fore-reef sites near the Tareu Pass, three from reef-top stations near Motu Ahi and two from Teonehua. This cluster consists of 175 species of benthic foraminifera (including back-reef taxa). They are characterized by higher abundances of *Amphistegina lessonii* and other symbiont-bearing foraminifera (*Heterostegina depressa*, *Sorites orbiculus*, *Peneroplis* spp.). *Amphistegina lessonii* accounted for 43%, *Eponides repandus* for 5%, *Sorites orbiculus* for 4%, *Anomalinella rostrata* for 3% and *Heterostegina depressa* for 2% of all specimens recovered from these sites. Specimens of *Homotrema rubra* are also prominent within this cluster. Other smaller foraminifera accounted for 19%.

#### Cluster D (Mangroves)

Cluster D is associated with coastal mangrove sites from around Teonehua. The foraminiferal assemblages of this cluster are characterized by higher abundances of *Quinqueloculina* and several stress-tolerant taxa. Quinqueloculinids constitute more than 60% at these sites followed by *Ammonia tepida* (17%), *Elphidium advenum* (5%), *Elphidium clavatum* and *Bolivina striatula* (2% respectively). *Peneroplis pertusus* is the only symbiont-bearing taxon present at these sites. The cluster houses a diverse assemblage of 41 species of benthic foraminifera. All of these foraminifera have come to live together in the mangroves since the plants were introduced to Moorea in 1937. The foraminifera were not introduced with the mangroves as all of them live in other assemblages elsewhere on Moorea. In that sense, Cluster D is not a natural assemblage with a long history at Moorea.

#### Cluster E (Inner Bay)

Cluster E is associated with the shallow inner inlets of Opunohu and Cook’s Bays. Opportunistic taxa constitute the majority of individuals recorded in this cluster (Tables 1 and 2). *Ammonia tepida* (12%), *Bolivina striatula* (13%), *Quinqueloculina* cf. *Q*. *semireticulosa* (9%), *Elphidium advenum* (6%) and *Nonionoides grateloupi* (3%) are among the most prominent in this habitat. *Quinqueloculina* is represented by 48 species and they constitute 25% of all specimens. Species of *Bolivina* make up 16%, while species of *Ammonia* and *Elphidium* are present with 12% and 11% respectively.

#### Cluster F (Fringing reefs)

Most of the samples in this cluster come from coastal fringing reef sites present in Opunohu and Cook’s Bays and fringing reef sites near Teonehua. The assemblages within this cluster are characterized by the presence of larger foraminifera including *Borelis schlumbergeri*, *Amphistegina lessonii*, *Peneroplis pertusus* and *Heterostegina depressa*. Smaller miliolids are particularly abundant with *Quinqueloculina* accounting for 19% of the total assemblage. The agglutinated species *Textularia foliacea foliacea* and *T*. *foliacea oceanica* \accounted for 3% and 2% respectively. *Hauerina pacifica* is the most abundant species within this cluster with 13% and is present at all sample sites. The cluster includes a total of 250 species of benthic foraminifera.

### Principal Component Analysis (PCA)

**Fig 7:**
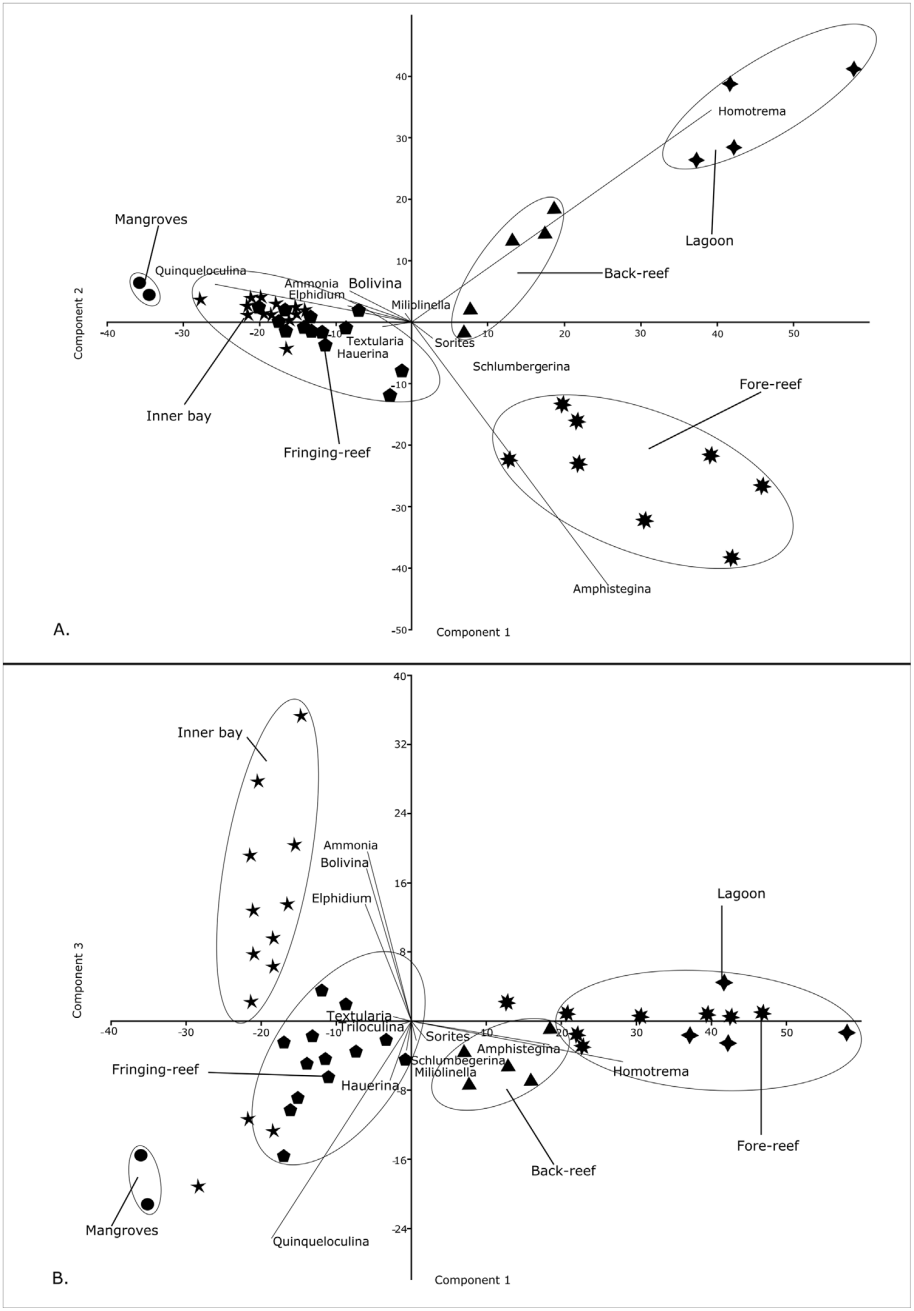
Principal Component Analysis (PCA) of the foraminiferafauna showing a) principal components 1 and 2, and b) principalcomponents 1 and 3. The symbols represent the clusters as defined in Fig 5.

The principal component analyses, based on percent abundance data of the 13 most frequent genera (which make up ~75% of the total population of foraminifera counted; Table 3), revealed a separation of two major habitats along the axis (Fig 7A). The first habitat group includes assemblages from fore-reef, back-reef and lagoon sites and the second is associated with taxa from the inner bays, mangroves and fringing reefs. The taxa are shown as vectors and their lengths represent the importance of individual genera as calculated by their eigenvalue. While the reefal sites are characterized by amphisteginids, *Homotrema* and *Sorites*, the nearshore mangrove, bay and fringing reef sites are dominated by the abundance of smaller miliolids and stress tolerant taxa like *Ammonia*, *Elphidium* and *Bolivina*. The *Amphistegina* vector is strongly related to the reefal sites and Clusters A, B and C where they represent the most abundant symbiont-bearing larger foraminifera. *Quinqueloculina* was the most abundant genus in nearshore mangrove settings and at some inner bay inlet sites. Samples from the inner Cook’s Bay and the mangrove sites at Teonehua contain abundant *Quinqueloculina* up to 51%. At Opunohu Bay, quinqueloculinids reach values of 35%. A similar pattern emerged when factor 1 and 3 were considered (Fig 7B) showing that assemblages differ along two major axis.

**Table 3:** Abundance records of selected genera of benthic foraminifera included in the Principal Component Analyses (PCA). Amm, *Ammonia*; Amp, *Amphistegina*; Bol, *Bolivina*; Elph, *Elphidium*; Hau, *Hauerina*; Hom, *Homotrema*; Mil, *Miliolinella*; Pen, *Peneroplis*; Quin, *Quinqueloculina*; Schl, *Schlumbergerina*; Sor, *Sorites*; Tex, *Textularia*; Tri, *Triloculina*.

Sample sites	Amm.	Amp.	Bol.	Elph.	Hau.	Hom.	Mil.	Pen.	Quin.	Schlu.	Sor.	Tex.	Tri.
M1	5	47	1	1	16	302	2	21	23	7	13	4	3
M3	5	32	4	10	7	211	9	5	26	4	5	1	0
M4	5	23	0	0	1	228	1	0	2	0	5	0	0
M7	67	3	13	48	19	0	0	18	332	0	1	0	4
M8	88	0	2	16	3	0	1	4	200	0	0	0	0
M9b	0	137	0	0	0	78	0	0	0	0	7	0	0
M10	2	0	13	52	53	0	5	11	78	0	2	0	0
M11	9	2	11	30	74	0	15	7	62	0	2	5	0
M13	59	2	33	40	13	0	5	0	60	0	2	2	14
M15	98	0	42	59	7	0	5	3	143	1	0	3	8
M16	9	55	4	13	3	62	13	7	70	16	7	2	4
M31	4	2	10	27	55	3	52	24	36	2	7	4	10
M32	6	2	0	2	118	0	28	25	66	5	11	6	36
M35	1	3	6	4	10	112	17	17	22	4	10	0	11
M40	125	0	109	126	0	0	0	0	84	0	0	18	0
M41	27	0	77	44	0	0	0	0	78	0	0	6	3
M43	5	0	48	124	0	0	0	0	58	0	0	4	0
M45	10	3	10	32	17	0	11	7	49	5	7	67	11
M47	28	0	133	9	0	0	2	0	49	0	0	20	1
M48	1	1	12	3	33	0	2	5	61	2	2	17	10
M49	2	3	9	6	47	0	4	7	96	12	8	24	19
M50	9	0	79	10	3	0	6	0	100	0	0	24	6
M51	5	0	49	0	0	295	0	0	8	0	0	0	0
M52	3	5	0	9	42	0	5	3	41	29	6	28	7
M53	0	8	0	1	7	90	30	10	40	48	18	0	1
M54	2	21	2	0	15	115	19	7	50	0	13	1	1
M56	2	0	22	22	2	0	19	5	180	0	0	4	15
M57	1	1	1	0	51	3	14	7	96	8	6	0	5
M58	0	43	1	6	1	76	12	4	75	3	35	0	0
M61	2	247	0	0	0	84	0	4	7	0	18	0	0
M62	0	134	2	1	6	51	1	6	30	2	27	0	1
M69	6	0	0	5	27	18	29	5	45	24	6	42	35
M71	2	173	0	0	0	105	1	0	6	24	23	0	0
M72	0	132	0	0	13	72	7	12	34	7	41	1	4
M75	178	0	9	24	0	0	2	0	18	0	0	0	15
M77	9	3	10	27	7	0	31	2	128	0	2	6	11
M78	12	0	79	14	0	0	0	0	69	0	0	16	20
M79	2	0	70	27	0	0	0	0	78	0	0	28	16
M83	6	22	20	4	16	0	2	2	111	1	0	2	16
M86	3	51	1	0	30	17	4	9	57	22	15	27	29
M87	1	6	18	8	10	9	14	6	113	3	5	40	4
M88	1	60	5	13	61	1	4	13	54	38	22	25	1
M93	0	127	0	0	3	130	6	3	22	7	7	0	5
M95	0	251	0	0	1	76	8	4	20	18	1	4	0
M96	0	101	0	1	4	64	6	5	20	10	4	1	3
Total	800	1700	905	818	775	2202	392	268	2997	302	338	432	329

### Ternary diagrams

**Fig 8:**
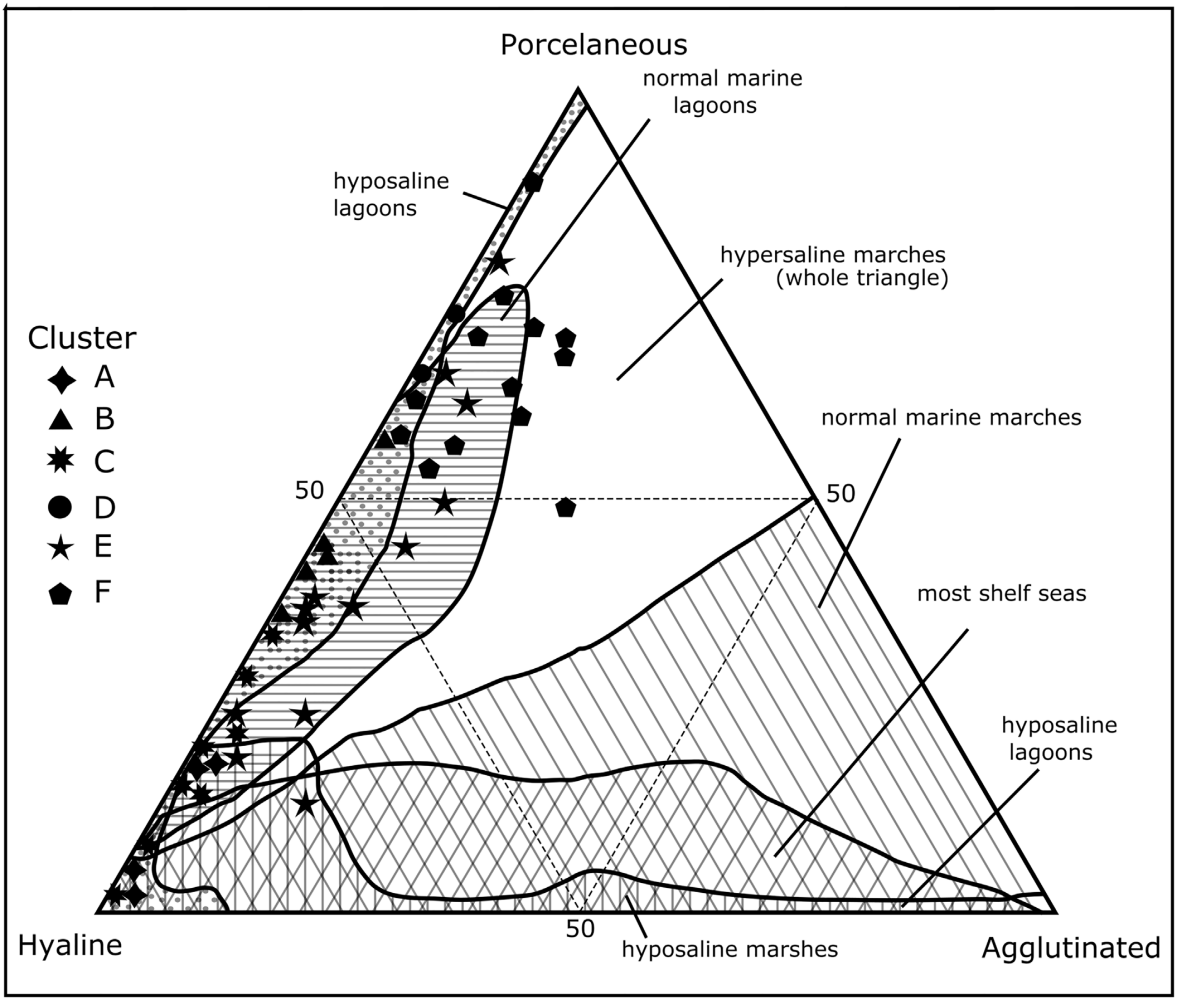
Ternary diagram showing percent abundances of wall structural types (porcellaneous, hyaline-perforate and agglutinated foraminifera) of individual sample sites around Moorea [55]. The symbols represent the clusters groups as defined in Fig 5.

As an independent line of evidence, percent abundances of wall structural types were calculated for each site and plotted in a standard ternary diagram (Fig 8). The resulting graph shows that percent abundances of wall structural types fall into two site-specific groups with back- and fore-reef and lagoon sites dominated by hyaline perforate taxa which contain few agglutinated species. These environments are equivalent to the sample sites present in Clusters A, B and C. The diagram further shows that samples from the inner bay, the fringing reefs and mangrove areas are generally characterized by a higher percentage of porcellaneous miliolids and a larger numbers of agglutinated specimens. Within the environmental fields of standard ternary diagrams provided by Murray [55], the mangrove sites fall within the hyposaline lagoon while the fringing reefs and the bay inlets mostly plot at the upper end of a normal marine lagoon. The agglutinated taxa have their highest numbers within the inner bays and the fringing reef areas of Opunohu and Cook’s Bays and represent the sites that are associated with Clusters E and F. Generally the clusters range between a normal marine and a hyposaline lagoon with a few samples outside the normal range. This is typical of warm tropical reefal and lagoonal settings [55].

### FORAM-Index (FI)

**Fig 9:**
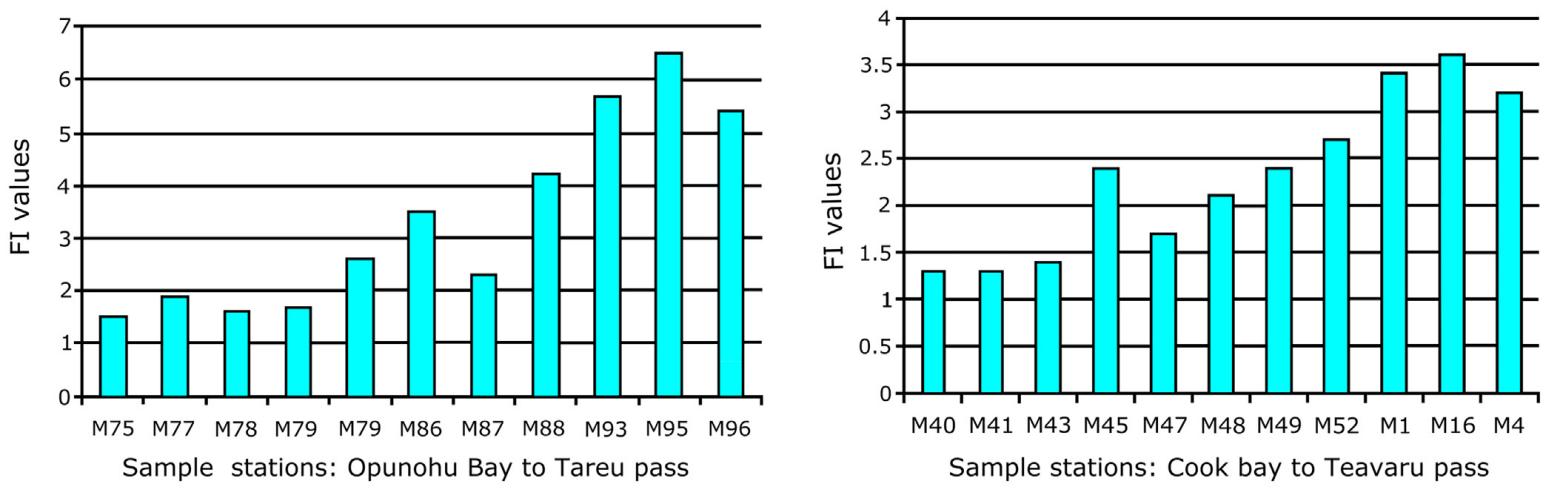
FI values plotted along transects in Opunohu and Cook’s Bays showing indices rising from the innermost bays towards the reefal sites.

In our samples the average FI in sediments was 3.1 ± 1.3. In Cook’s Bay, the average FI value is comparatively low (2.1), at least in the innermost bays, and it is dominated by stress-tolerant opportunistic taxa such as *Ammonia*, *Bolivina*, *Elphidium*, and *Nonionoides*. Some symbiont-bearing taxa were present including *Amphistegina lessonii*, *Peneroplis pertusus* and *Sorites orbiculus*. At a few sites in Cook’s Bay the larger benthic foraminifera constitute up to 9 percent of the samples (M11, M35, M49, M52). FI values lower than 2, indicating an environment not suitable for reef accretion [31], were common in the innermost parts of both Cook’s and Opunohu Bays and at nearshore sites around Teonehua. In Opunohu and Cook’s Bays FI values gradually increase from the innermost parts to the fringing reefs and to the fore-reefs (Fig 9). Calculation of the FI values around the reef-top and the fore-and back-reef areas revealed much higher values ranging from 5.4 to 6.5 at Tareu, from 6.6 to 6.9 at Motu Ahi, and up to 7.7 at Teonehua.

## Discussion

### Foraminifera Diversity

Coral reefs and lagoons of the western Pacific Ocean contain extremely diverse assemblages of benthic foraminifera [9, 44, 60, 66]. The reefal environments of Moorea also harbor particularly diverse assemblages of benthic foraminifera that rival those found elsewhere in the Indo-Pacific except for the absence of certain large symbiont-bearing taxa. At least 422 species belonging to agglutinated, perforate-hyaline and imperforate-porcellaneous groups, including some larger symbiont-bearing taxa, occur on the island. This species richness more than doubles the number of taxa previously documented from Moorea. The number includes 380 species found in our study and an additional 42 from Langer and Lipps [9]. In a study that formed an integral part of this research project, Vénec-Peyré (41) identified within the French Polynesian Islands 182 species belonging to 39 families of foraminifera [41].

The total number of benthic foraminifera around Moorea is significantly higher than the number of corals reported from French Polynesia at large [23,67,68], and this is likely due to the large number of habitats sampled during this campaign. The shallow nearshore habitats around mangrove trees contain almost 100 species [9], indicating that the waters of the Society archipelago harbor a wealth of species that remain to be discovered. Among the total of 380 species are 130 hyaline perforate, 217 porcellaneous imperforate and 33 agglutinated taxa. While 217 species of porcellaneous miliolids occur at Moorea, only 101 species are present in the Papuan lagoon system [66]. The greater abundance of miliolids at Moorea is far higher than expected considering the location of the lagoon at Papua New Guinea in the heart of the hotspot of diversity in the coral triangle [44,69]. No calcarinids, which are common constituents of the highly diverse coral triangle environments (see also [8,41,70–73]), are present at Moorea.

The diversity of foraminiferal biotas is reflected in high Fisher *α* diversity indices. These values are highest around reefal sites, in particular within the fringing reefs, where bay and reefal biotas occur together. The Fisher *α* index generally increases in the number of species from the inner bay towards the open ocean (Fig 4), thus confirming a trend that was previously recognized along transects in the lagoon at Madang, Papua New Guinea [44,9]. Species richness along transects at Moorea also increases from the shore towards the reef barrier, but individual sites vary considerably [8]. Similar patterns of distribution around Moorea were also observed for macrophytes [74], fish [75] and molluscs [76]. The highest number of species-77, are in front of the fringing reefs at the outer margins of the two major bays. There, foraminifera from organic-rich inner bay sites, mangroves, *Paspallum* and *Hibiscus* habitats in addition to fringing reefs and channel habitats amalgamate. Therefore, we attribute this to the imbrications of habitats and amalgamation of biotas that occurs along this part of the bays. At Moorea, the richest environments occur in those areas that offer a greater variety of biotopes [8].

Foraminiferal assemblage composition differed significantly among habitats. At the reefal sites (Clusters A, B and C), *Amphistegina lessonii* is the dominant taxon with abundances of up to 64%. They are particularly prominent at the fore-reef sites of Terau Pass at the entrance of Opunohu Bay, a site that is typical of other fore-reef habitats on the island [8]. Because of their abundance, ubiquity, significant carbonate production and ability to modify the composition of carbonate sediments, amphisteginid foraminifera are considered environmental engineers [77]. Amphisteginids domination in reefal environments [8,72,78,79] may be due to their ability to tolerate higher wave energy. Other species frequently found in reefal habitats were *Homotrema rubra*, *Anomalinella rostrata*, *Eponides repandus*, *Heterostegina depressa*, with few *Schlumbergerina alveoliniformis*, *Sorites orbiculus* and *Peneroplis pertusus*. *Schlumbergerina alveoliniformis* was more abundant in back-reef habitats, and thus exhibit environmental preferences that were also reported at Tahiti [72]. Apparently, some of these taxa flourish particularly well in reefal sites [42, 44, 60, 69]. As such, the foraminiferal assemblages from reefs at Moorea share similar distributions to those of other western Pacific tropical islands. As very good indicators of reefal habitats, they preserve ecologic information useful in comparative analyses over decadal periods and long-term paleoecological studies.

*Quinqueloculina*, with over 90 species, strongly affect the configuration of sites within the cluster groups (Fig 6) forming the bulk of the lagoonal and nearshore assemblages (M7-8, M15, M49, M50, M56, M83, M87). Clusters E and F show numerically abundant and similar proportions of *Quinqueloculina* indicative of back-reef lagoonal habitats [55]. *Quinqueloculina seminula* and *Q*. cf. *Q*. *semireticulosa* are the most abundant miliolids, and they occur in almost all samples, especially within Opunohu and Cook’s Bays. The miliolids in total accounted for 45% of all the foraminifera in the lagoon. This pattern is similar to other Pacific islands and other Indo-Pacific reef-lagoonal settings [60,80].

Agglutinated taxa accounted for 4% of all the foraminifera counted. *Textularia foliacea foliacea* and *T*. *foliacea oceanica* are most abundant in the fringing-reef and bay habitats and both accounted for 1.2% of the total foraminifera assemblage. Symbiont-bearing taxa generally decrease in the abundance from the reef sites towards the lagoon and the inner bay habitats.

Stress tolerant species of *Bolivina*, *Ammonia*, *Elphidium* and *Nonionoides* occur in greater numbers within inner portions of the mangrove-surrounded bay inlets that are covered by dark fine-grained and low-oxygen sediments (up to 69%). These species are typical of hypo- to normal salinity lagoons [31,81–83]. Dark, organic-rich sediments dominate the inner bays creating ideal conditions for such assemblages, probably because of increased numbers of bacteria. Anthropogenic activities including sewage disposal, fish farming, and uncontrolled tourism contribute to an expansion of these areas. To what degree individual factors control specific abundances and the composition of the inner bay assemblages remains to be determined. The specificity of inner bay foraminiferal associations is, however, important for monitoring ecologic changes and reconstruction of paleoenvironments.

At both Moorea and Madang (PNG), larger symbiont-bearing foraminifera and agglutinated species are either extremely rare or absent in the innermost harbor and bays where species of *Ammonia* and *Elphidium* constitute almost the entire foraminiferal fauna [44]. However, in Cook’s and Opunohu Bays, *Bolivina* and *Nonionoides* are abundant; these two taxa tolerate low-oxygen conditions in sediments rich in organic material [31,84,85].

The cluster diagram (Fig 5) revealed a distribution that centers on environmental factors characterizing individual habitats and substrate types. Sites with similar conditions are grouped together and tend to harbor assemblages dominated by specific species and genera. Concentration ratios quantified these observations as demonstrated by the Principal Component Analysis (PCA) and ternary plot analyses (Figs 7 and 8). Thick-shelled, symbiont-bearing taxa with robust tests, including *Amphistegina*, *Sorites*, *Parasorites* and *Heterostegina*, accounted for the largest proportions in reefal habitats. *Amphistegina* had the highest concentration of the larger benthic foraminifera near reef-top and back- and fore-reef sites. Robust tests are particularly resistant to abrasion and enhance their accumulation in carbonate environments [86].

Miliolid species of *Quinqueloculina* were found in all habitats but abundances were notably higher in near-shore and lagoonal environments characterized by phytal vegetation. The dominance of smaller non-symbiont bearing miliolids in phytal substrates also occur in lagoonal habitats of Scilly Atoll [5], Papua New Guinea [44,69], New Caledonia [42,72,82], Bazaruto, E-Africa [87] and the Caribbean [88].

Each cluster group contains numerically abundant indicator species or genera that do not occur in high abundances in other faunal clusters. This implies low horizontal transport rates within the reef, lagoon and bay habitats, and signifies that faunal mixing among the cluster groups is limited. Foraminiferal death assemblages are mostly autochthonous and thus preserve environmental information that is useful in paleoecologic and ecologic interpretations.

### FORAM-Index (FI)

The FORAM Index, based on total assemblages, indicate a general rising FI towards the reef barrier as reflected in the abundance and taxonomic richness of larger symbiont-bearing foraminifera. Highest total species richness values occur, however, at the fringing reefs along the outer margins of the two major bays due to imbrication of foraminiferal habitats from nearshore, reefal, mangrove, bay channel and fringing reefs.

The FI of total assemblages from Moorea accord with live reef assemblages [89–92], indicating that the water quality at most back- and fore-reef sites supports calcifying symbiosis and suitable for reef carbonate accretion. Moderate to low FI were recorded at nearshore and lagoonal sites of the N-coast between Irihoriu Pass and Teavaru (FI 2.0–3.6). These areas have low coral cover and branching corals with smaller colonies, probably resulting from greater anthropogenic impacts [4].

Moorea underwent severe bleaching events in 1982, 1983 and every 2–5 years since 1991 [20,93] as well as natural disturbances resulting in spatio-temporal heterogeneity in coral reef cover and recruitment [20,22,23,25,27–29], although the reefs recovered in 10 to 12 years [13,14]. The FI indicate that most fringing reefs and back and fore-reef sites are favorable for reef growth. The low FI recorded at the innermost bays sites reflect both the dark organic-rich, fine grained substrate and the coverage of mangrove stands. In addition, small rivers enter the bays at their southern ends, form small delta-like fans, and are sources of agricultural, sewage and nutrient runoff. At least in Opunohu Bay, the FI rise to higher levels at the outer bay margin, while at Cook’s Bay, the values indicate a continuous impact on the composition of foraminiferal assemblages. Increased runoff, nutrient loading, reef destruction, and a future rise of tourism in these areas will certainly be of concern, impacting reefal growth outside the bays and possibly affecting carbonate accretion of the reef barrier protecting the island of Moorea. These impacts will be enhanced by climate warming, ocean acidification and sea level rise anticipated in the next several decades.

## Conclusions

This study together with those of Vénec-Peyré [8,41,46] constitutes the most extensive investigation yet of the foraminifera from shallow-water nearshore and reefal environments around Moorea. A total of 422 (380 from our study) species has been recorded, a number that more than doubles previously documented inventories of species counts. The benthic foraminifera around Moorea have large-scale spatial distribution patterns of habitat specific assemblages. These habitat preferences are also reflected in abundance patterns of individual species, genera and functional groups. Diversity gradients generally increase from bay inlets to the reef barrier, but highest species richness is in fringing reefs, an area that represents a mosaic of habitats.

The abundance of functional groups of foraminifera (symbiont- bearing, heterotrophic, opportunistic) together with Foraminiferal Index (FI) calculations identified environments suitable and critical to support calcifying symbiosis and carbonate accretion. FI indicate that the innermost bays and some outer bay fringing reef habitats are under direct natural and anthropogenic influences.

While Moorean reefal foraminifera deserve more scientific attention, particularly acquisition of more quantitative data, our findings are sufficient for monitoring rising influences of natural events and anthropogenic activities. Future changes can be compared with our baseline data from 1992 and the development of those changes over time can be determined by collecting dead specimens from particular time periods. In addition, as global warming, anoxia and acidification of the oceans increase, foraminifera can provide rapid indication of these world-wide changes as well as local ones such as pollution, impacts on reefs due to industrial development and tourist activities. Foraminifera are easily collected and the indices, diversity and abundances are easily determined in the laboratory.

## Supporting Information

S1 List Foraminiferal species in alphabetical order (*denotes additional species recorded by Langer and Lipps, 2006; **denotes additional species recorded by Vénec-Peyré, 1985). Species identified to generic level only are summarized under their generic name (spp.).
(DOC)
