# Supplementary material for: Spatial Patterns in the Distribution, Diversity and Abundance of Benthic Foraminifera around Moorea (Society Archipelago, French Polynesia)
Source: PLoS One. 2015 Dec 28;10(12):e0145752. doi: 10.1371/journal.pone.0145752 (PMC4692423; doi:10.1371/journal.pone.0145752)
Supplement: S1 List — Species identified to generic level only are summarized under their generic name (spp.). (DOC) [file pone.0145752.s001.doc]

**Supporting Information (S1)**

List of foraminiferal species in alphabetical order (*denotes additional species recorded by Langer and Lipps, 2006; **denotes additional species recorded by Vénec-Peyré, 1985). Species identified to generic level only are summarized under their generic name (spp.).

*Abditodentrix rhomboidalis* (Millett 1899)

*Acervulina mabahethi* (Said 1949)

*Acervulina inhaerens* Schultze, 1854**

*Acupeina triperforata* (Millett 1899)

*Adelosina laevigata* d’Orbigny 1826

*Adelosina* sp.

*Agglutinella agglutinans* (d’Orbigny 1839)

*Ammoastuta salsa* Cushman & Brönnimann, 1948*

*Ammobaculites agglutinans* (d’Orbingy, 1846)**

*Ammobaculites exiguus* Cushman & Brönnimann, 1948

*Ammomassilina alveoliniformis* (Millett, 1898)**

*Ammonia convexa* Collins, 1958*

*Ammonia tepida* (Cushman, 1926)*

*Ammonia* cf*. A. tepida* (Cushman, 1926)*

*Ammoscalaria compressa* (Cushman & McCulloch, 1939)

*Ammotium* sp.*

*Amphisorus hemprichii* Ehrenberg, 1839

*Amphistegina gibbosa* d’Orbigny, 1839**

*Amphistegina lessonii* d’Orbigny, 1843

*Amphistegina lobifera* Larsen, 1976**

*Amphistegina quoyi* d’Orbigny, 1826**

*Amphistegina radiata* (Fichtel and Moll, 1798)**

*Amphistegina* sp.*

*Anomalina globulosa* Chapman and Parr, 1884**

*Anomalina? maculosa* Todd, 1957**

*Anomalinella rostrata* (Brady, 1881) *

*Anomalinoides* sp*

*Anomalinulla glabrata* (Cushman, 1924)

*Articulina alticostata* Cushman, 1944*

*Articulina pacifica* Cushman, 1944

*Articulina queenslandica* Collins, 1958

*Articulina sagra* d'Orbigny, 1839

*Assilina ammonoides* (Gronovius, 1781)

*Baggina phillipinensis* (Cushman, 1921)

*Bolivina compacta* Sidebottom, 1905**

*Bolivina currai* Selier de Civrieux, 1976**

*Bolivina doniezi* Cushman & Wickenden, 1929

*Bolivina earlandi* Parr, 1950**

*Bolivina lowmani* Phleger and Parker, 1951**

*Bolivina rhomboidalis* Millett, 1895**

*Bolivina seminuda* Cushman, 1911**

*Bolivina spathulata* (Williamson, 1859)**

*Bolivina spinata* Cushman, 1937**

*Bolivina striatula* Cushman 1922*

*Bolivina sublitoralis* Cellier de Civrieux, 1976**

*Bolivina* cf*. B. suezensis* Said, 1949

*Bolivina torqueata* Cushman & McCulloch, 1942**

*Bolivina variabilis* (Williamson, 1858)

*Bolivinella elegans* Parr 1932

*Bolivinella folia* (Parker and Jones, 1865)**

*Brizalina* cf*. B. subspathulata* (Boomgaart, 1949) *

*Brizalina* sp*.* *

*Borelis schlumbergeri* (Reichel, 1937)

*Bulimina aculeta* d’Orbingy, 1826**

*Bulimina marginata* d’Orbingy, 1826**

*Bulimina* spp.

*Buliminella elegantissima* (d'Orbigny, 1839)

*Buliminella madagascariensis* d'Orbigny, 1826**

*Buliminella miletti* Cushman, 1939**

*Buliminoides williamsonia* (Brady, 1984)**

*Bronnimannia haliotis* (Heron-Allen & Earland, 1924)

*Calcarina calcar* d’Orbingy, 1826**

*Cancris auriculus* (Fichtel & Moll, 1798)

*Caronia exilis* (Cushman & Brönnimann, 1948)

*Carpenteria monticularis* (Carter, 1877)**

*Cerebrina lacunata* (Burrows & Holland, 1895)

*Cibicidella variabilis* (d’Orbingy, 1826)**

*Cibicides advenum* (d’Orbingy, 1826)**

*Cibicides mabaheti* Said, 1949

*Cibicides mayori* (Cushman, 1924)

*Cibicides pseodulobatulus* Perelis and Reiss, 1975*

*Cibicides pseudoungerianus* (Cushman, 1922)**

*Cibicides* cf*. C. refulgens* de Montfort, 1808

*Cibicides* spp.

*Cibrobaggina reniformis* (Heron-Allen & Earland, 1915)

*Clavulina difformis* Brady, 1884

*Clavulina cf. C. multicamerata* Chapman, 1907

*Clavulina pacifica* Cushman, 1924

*Clavulina* sp.

*Clavulina tricarinata* d’Orbingy, 1839**

*Clavulina angularis* d’Orbingy, 1826**

*Clavulinoides* sp.*

*Conicospirillinoides* sp. Cheng & Zheng, 1978

*Cornuspira foliacea* (Philippi, 1844),

*Cornuspira planorbis* Schultze, 1853. *

*Cornuspira selseyensis* Heron-Allen & Earland, 1909

*Coscinospira hemprichi* Ehrenberg, 1839

*Cribroelphidium poeyanum* (d’Orbingy, 1839)**

*Cyclammina subtrullisata*, (Parr, 1950)

*Cycloclypeus carpenteri* Brady, 1881**

*Cyclogyra involvens* (Reuss, 1849)**

*Cycloforina* cf. *C. carinata* (Albani 1974) *

*Cycloforina* cf*. C. granulocostata**

*Cymbaloporella tabellaeformis* (Brady, 1884)**

*Cymbaloporetta bermudezi* (Sellier & Civrieux, 1976) *

*Cymbaloporetta bradyi* (Cushman, 1924)

*Cymbaloporetta sauammosa* (d’Orbingy, 1839)**

*Cymbaloporetta* spp.

*Chrysalidinella dimorpha* ( Brady, 1881)**

*Discorbinella* cf. *D. bertheloti* (d'Orbigny, 1839)

*Discorbinella* sp.*

*Discorbis* cf*. D. mira* Cushman, 1922*

*Dyocibicides* sp Cushman & Valentine, 1930

*Eggerelloides* sp.

*Elongobula milletti* (Cushman, 1933)

*Elongobula* *spicata* (Cushman & Parker, 1942)

*Elongobula* *parallela* (Cushman & Parker, 1931)

*Edentostomina* *cultrata* (Brady 1881) *

*Edentostomina* spp.

*Elphidium* *advenum* (Cushman, 1922)

*Elphidium* cf*. E. articulatum* (d'Orbigny, 1839)*

*Elphidium* *botaniensis* Albani, 1981

*Elphidium* *clavatum* Cushman, 1930

*Elphidium craticulatum* (Fichtel & Moll, 1798)*

*Elphidium* cf. *E*. *excavatum* Cushman, 1930

*Elphidium* *oceanicum* Cushman, 1933

*Elphidium* *lene* Cushman & McCulloch, 1940

*Elphidium* cf. *E. crispum* (Linnaeus, 1758)

*Elphidium* *maorium* Hayward, 1997

*Elphidium milletti* (Heron-Allen & Earland, 1915)

*Elphidium simplex* Cushman, 1933

*Elphidium williamsoni* Haynes, 1973*

*Elphidium* cf*. E. williamsoni* Haynes, 1973

*Elphidium* spp.

*Epistominella tubulifera* (Heron-Allen and Earland, 1915)**

*Eponides* *repandus* (Fichtel & Moll, 1798) *

*Eponides* sp.

*Euthymonacha* *polita* (Chapman, 1900)

*Falsagglutinella* *angularis* Loeblich & Tappan, 1994

*Fischerinella* *diversa* McCulloch, 1977

*Fijiela* *simplex* (Cushman, 1929)

*Fissurina* *bispinata* Ujiié, 1963

*Fissurina* *lacubrata* (Burrows and Holland, 1895)**

*Fissurina* *cf. F. tuberculata* Brady**

*Fissurina* *squammoso-marginata* Brady, 1884**

*Fissurina* spp.

*Fursenkoina* *schreibersiana* (Czjzek, 1848)

*Fursenkoina* sp*.**

*Gaudryina cf. G. collinsi* Cushman, 1936**

*Gaudryina triangularis angulata* Cushman, 1924**

*Gavelinopsis* sp Hofker, 1951

*Glabratella erecta* (Sidebottom, 1908)**

*Glabratella globosa* (Sidebottom, 1909)**

*Glabratella makinoi* Uchio, 1952**

*Glabratella patelliformis* (Brady, 1884)**

*Globocassidulina* *crassa* (d'Orbigny, 1839)

*Globocassidulina* *subglobosa* (Brady, 1881)

*Gypsina vesicularis* (Parker & Jones, 1860) *

*Haddonia* sp. Chapman, 1898

*Haynesina depressula* (Walker & Jacob, 1798)

*Hauerina bradyi* Cushman, 1918**

*Hauerina compressa* d’Orbingy, 1846**

*Hauerina diversa* Cushman, 1946

*Hauerina fragilissima* (Brady, 1884)

*Hauerina ornatissima* (Karrer, 1868)

*Hauerina pacifica* Cushman, 1917*

*Heterostegina depressa* d'Orbigny, 1826

*Heterostegina* cf. *H*. *curva* Moebius, 1880

*Heterostegina* sp. *

*Homotrema rubra* (Lamarck, 1816)

*Hopkinsina victoriensis* Collins, 1974

*Hopkinsinella glabra* (Millett, 1903)

*Lagena desmophora* Jones, 1872**

*Lagena filicusta* Reuss, 1862**

*Lagena spicata* Cushman & McCulloch, 1950

*Lagena spiralis* Brady, 1884**

*Lagena striata* d‘Orbingy, 1839**

*Lagena strumosa* Reuss, 1858

*Lenticulina* sp.

*Lituotuba minuta* Collins, 1958*

*Lituotuba sp.**

*Lobatula lobatula* (Walker & Jacob, 1798)

*Loxostomina* cf*. L. africana* (Smitter, 1955) *

*Loxostomina limbata* (Brady, 1881)

*Loxostomina mayori* (Cushman, 1922)

*Loxostomum convalarium* (Millett, 1900)**

*Massilina crenata* (Karrer, 1868)**

*Massilina granulocostata* (Germeraad, 1946)

*Massilina inaequalis* Cushman, 1921

*Mesosigmoilina minuta* (Zheng, 1979)

*Miliola sublineata* (Brady, 1884)

*Miliolinella australis* (Parr, 1932)

*Miliolinella heligmateira* Loeblich & Tappan, 1994

*Miliolinella labiosa* (d'Orbigny, 1839)

*Miliolinella* cf. *M. labiosa* (d'Orbigny, 1839)

*Miliolinella oceanica* (Cushman, 1932)

*Miliolinella* cf. *M. oceanica* (Cushman, 1932) *

*Miliolinella pilasensis* McCulloch, 1977

*Miliolinella subrotunda* (Montagu, 1803)

*Miliolinella* spp.

*Millettia tesselata* (Brady, 1884)**

*Millettiana milletti* (Heron-Allen & Earland, 1915) *

*Miniacina miniacea* (Pallas, 1766)**

*Monalysidium acicularis* (Batsch, 1791)

*Monalysidium confusa* (McCulloch, 1977)

*Monalysidium* sp.

*Mimosina* *histrix* Millett, 1900

*Mimosina* sp.

*Murrayinella murayyi* (Heron-Allen & Earland, 1915)

*Neoconorbina albida* McCulloch, 1977

*Neoconorbina terquemi* ( Rhzehak, 1888)**

*Neoconorbina tuuberocapitula* (Chapmann, 1889)**

*Neoconorbina* spp.

*Nodophthalmidium* *antillarum* (Cushman, 1922)

*Nonionoides* *grateloupi* (d'Orbigny, 1826)

*Nonion sloani* (d'Orbigny, 1839)**

*Nonion* sp. 1

*Nonion* sp. 2

*Nubeculina* *advena* Cushman, 1924

*Oolina striatopunctata* (Parker and Jones, 1923)**

*Oolina* sp.

*Palliolatella* *fasciata* *carinata* (Sidebottom, 1906)

*Parasorites orbitolitoides* (Hofker, 1930)

*Parasorites* cf*. P. orbitolitoides*

*Paratrochammina simplissima* (Cushman & McCulloch, 1948)*

*Paratrochammina* cf*. P. simplissima* (Cushman & McCulloch, 1948)

*Paratrochammina stoeni* Brönnimann & Zaninetti, 1979 *

*Paratrochammina* sp.

*Parrellina milletti* (Heron-Allen & Earland, 1915) *

*Parrina bradyi* (Millett, 1898)**

*Patellina corrugata* Williamson, 1858**

*Pitella haigi* Langer, 1992*

*Pitella transversestriata* (Brady 1881)*

*Peneroplis pertusus* (Forskål, 1775)

*Planogypsina squamiformis* (Champman, 1901)**

*Planispirillina decorata* Brady, 1884**

*Planispirillina papillosa* (Cushman, 1913)**

*Planispirillina spinigera* (Chapman, 1900)

*Planispirillina* cf. *P. tuberculatolimbata* (Chapman, 1900)

*Planispirinella exigua* (Brady, 1879)**

*Planispirinella involuta* Collins, 1958

*Planogypsina acervalis* (Brady, 1884)*

*Planorbulinella larvata* (Parker and Jones, 1865)**

*Planorbulinoides reticanulatus* (Parker and Jones, 1862)**

*Poroeponoides lateralis* (Terquem, 1878) *

*Proroeponides cribrorepandus* Asano and Uchio, 1951**

*Porosononion* sp.

*Procerolagena oceanica* (Albani, 1974)

*Pseudogaudryina* sp.

*Pseudohauerinella dissidens* (McCulloch, 1977)

## Pseudohauerina orientalis (Cushman, 1946)

## Pseudomassilina cf. P. australis **(Cushman, 1932)**

*Pseudononion granuloumbilicatum* Zheng, 1979

*Pseudononion* sp.

*Pseudoschlumbergerina ovata* (Sidebottom, 1904)

*Pseudotriloculina subgranulata* (Cushman, 1918)

*Pseudotriloculina* sp.

*Pyrgo canariensis* (d'Orbigny, 1839)**

*Pyrgo denticulata* (Brady, 1884)**

*Pyrgo elongata (*d'Orbigny, 1826)**

*Pyrgo oblonga* (d'Orbigny, 1839)*

*Pyrgo striolata* (Brady, 1884)*

*Prygo* spp.

*Quinqueloculina agglutinans* d'Orbigny, 1839

*Quinqueloculina* *angulariformis* McCulloch, 1977

*Quinqueloculina arenata* Said, 1949

*Quinqueloculina barnadi* Rasheed 1971*

*Quinqueloculina bicarinata* d'Orbigny, 1826*

*Quinqueloculina bosciana* d'Orbigny, 1839**

*Quinqueloculina bradyana* Cushman, 1917

*Quinqueloculina collumnosa* Cushman, 1922

*Quinqueloculina corrugata* (Collins, 1958)*

*Quinqueloculina crassicarinata* Collins, 1958

*Quinqueloculina cuvieriana* d'Orbigny, 1839

*Quinqueloculina* *debenayi*, Langer 1992

*Quinqueloculina* cf*. Q. debenayi* Langer, 1992

*Quinqueloculina deliculata* Vella, 1957

*Quinqueloculina disparilis* d'Orbigny, 1826

*Quinqueloculina distorqueata* Cushman, 1954

*Quinqueloculina* cf. *Q distorqueata* Cushman, 1954

*Quinqueloculina eburnea* (d'Orbigny, 1839)

*Quinqueloculina exmouthensis* Parker, 2009

*Quinqueloculina exsculpta* (Heron-Allen & Earland, 1915)

*Quinqueloculina ferox* (Rhumbler, 1907)**

*Quinqueloculina funafutiensis* (Chapman, 1900)

*Quinqueloculina* *granulocostata* Germeraad, 1946

*Quinqueloculina* cf. *Q. granulocostata* Germeraad, 1946

*Quinqueloculina incisa* Vella, 1957

*Quinqueloculina* cf*. Q. incisa* Vella, 1957*

*Quinqueloculina* cf*. Q. incisura* Todd 1957

*Quinqueloculina jugosa* Cushman, 1944

*Quinqueloculina lamarckina* d'Orbigny, 1839**

*Quinqueloculina latidentella* Loeblich & Tappan, 1994*

*Quinqueloculina lizardi* Baccaert, 1987

*Quinqueloculina neostriatula* Thalmann, 1950

*Quinqueloculina* cf. *Q. oblonga* (Montagu, 1803)

*Quinqueloculina parkeri* (Brady, 1881)

*Quinqueloculina parvaggluta* Vella, 1957

*Quinqueloculina philippinensis* Cushman, 1921

*Quinqueloculina poeyana* d'Orbigny, 1839

*Quinqueloculina polygona* d'Orbigny, 1839

*Quinqueloculina pulchella* d'Orbigny, 1826

*Quinqueloculina quinquecarinata* Collins, 1958

*Quinqueloculina* cf*. Q. rugosa* d'Orbigny, 1839

*Quinqueloculina samoensis* Cushman, 1924**

*Quinqueloculina seminula* (Linne 1767)

*Quinqueloculina cf. Q. semireticulosa* Cushman, 1932

*Quinqueloculina sidebottomi* (Rasheed, 1971)

*Quinqueloculina* cf. *Q. socorroensis* McCulloch, 1977

*Quinqueloculina stelligera* Schlumberger, 1893

*Quinqueloculina subcuneata* Cushman, 1921

*Quinqueloculina subulosa* Cushman, 1942**

*Quinqueloculina subparkeri* McCulloch, 1977

*Quinqueloculina subpolygona* Parr, 1945

*Quinqueloculina sulcata* d’Orbingy, 1826**

*Quinqueloculina tantabiddyensis* Parker, 2009

*Quinqueloculina tricarinata* d’Orbingy, 1839**

*Quinqueloculina tropicalis* Cushman, 1924

*Quinqueloculina vandiemeniensis* Loeblich & Tappan, 1994

*Quinqueloculina viennensis* Le Calvez, 1958**

*Quinqueloculina zhengi* Parker, 2009

*Quinqueloculina* spp*.*

*Rectoglandulina* sp*.**

*Reophax* cf*. R. bacillaris* Brady, 1881*

*Reophax communis* Lacroix, 1930

*Reophax irregularis* Parker, 1954

*Reophax* scorpiurus Montfort, 1808**

*Reophax* sp.

*Reusella pacifica* Cushman and McCulloch, 1948

*Reusella simplex* Cushman, 1929**

*Reusella spinulosa* (Reuss, 1850)**

*Rhabdammina* sp.

*Rosalina advena* (Cushman, 1931)**

*Rosalina bradyi* (Cushman, 1915)*

*Rosalina concinna* (Brady, 1884)**

*Rosalina globularis* d'Orbigny, 1826

*Rosalina orbicularis* Terquem, 1876**

*Rosalina* sp.

*Rotorbis* cf*. R. auberii* (d'Orbigny, 1839)

*Rotorbis* sp.

*Rotorboides granulosus* (Heron-Allen & Earland, 1915)

## Sagrinella convallaria (Millett, 1900)

## Sagrinopsis fimbriata(Millett, 1900)

*Sahulia* sp.

*Saidovina cf. S. carinata* (Millett, 1900)

*Schlumbergerina alveoliniformis* (Brady, 1879)

*Septotextularia rugosa* Cheng & Zheng, 1978

*Sigmavirgulina tortuosa* (Brady, 1881)

*Sigmohauerina bradyi* (Cushman, 1917)

*Sigmoihauerina involuta* (Cushman, 1946)

*Sigmoilinella* cf*. S tortuosa* Zheng, 1979

*Sigmoilinita costata* (Schlumberger, 1893)

*Sigmoilopsis elliptica* (Galloway & Wissler, 1927)

*Siphonaperta distorqueata* (Cushman, 1954)

*Siphonaperta pittensis* (Albani, 1974)

*Siphonaperta* cf*. S. pittensis* (Albani, 1974) *

*Siphonaperta wiesneri* (Parr, 1950) *

*Siphonaperta subagglutinata* (Asano, 1936)

*Siphogenerina raphana* (Parker & Jones, 1865)

*Siphogenerina striata* (Schwager, 1866)

*Siphogenerina virgula* (Brady, 1879) *

*Siphogenerina* sp.

*Siphonina tubulosa* Cushman, 1924

*Siphonaperta pittensis* (Albani, 1974)

*Siphoninoides echinatus* (Brady, 1879)**

*Siphotextularia crispata* (Brady, 1884)

*Sorites marginalis* Lamarck, 1816)**

*Sorites orbiculus* (Forskål, 1775)

*Sphaerogypsina globula* (Reuss, 1848)

*Spirillina grosseperforata* Zheng, 1979

*Spirillina* cf. *S. sigillata* McCulloch, 1977*

*Spirillina* spp.

*Spirolina arietina* (Batsch, 1791) *

*Spirolina pedum* d'Orbigny, 1826**

*Spiroloculina acescata* Cushman, 1932

*Spiroloculina angulata* Cushman, 1917

*Spiroloculina antillarum* d'Orbigny, 1839*

*Spiroloculina caduca* Cushman, 1922

*Spiroloculina communis* Cushman & Todd, 1944

*Spiroloculina* cf. *S. communis**

*Spiroloculina convexa* Said, 1949

*Spiroloculina corrugata* Cushman & Todd, 1944

*Spiroloculina exima* Cushman, 1922**

*Spiroloculina mayori* Cushman, 1924

*Spiroloculina caduca* Cushman, 1922

# *Spiroloculina ornata* d’Orbingy, 1839**

*Spiroloculina samoaensis* Cushman, 1924

*Spiroloculina scrobiculata* Cushman, 1921

*Spiroloculina* spp.

*Spirophtalmidium* cf*. S. eleganitissimum* (Said, 1949)*

*Spirophthalmidium prolixum* Loeblich & Tappan, 1994

*Spirophthalmidium scabrum* Loeblich & Tappan, 1994

*Spirophthalmidium* sp.

*Spirosigmoilina bradyi* Collins, 1958

*Spirosigmoilina parri* Collins, 1958

*Stictogongylus rugata* (Heron-Allen & Earland, 1928)

*Strebloides* sp.

*Svratkina australensis* (Chapman, Parr and Collins, 1934)**

*Textularia agglutinans* d'Orbigny, 1839

*Textularia candeiana* d'Orbigny 1839

*Textularia cushmani* Said, 1949

*Textularia earlandi Parker, 1952***

*Textularia foliacea foliacea* Heron-Allen and Earland, 1915

*Textularia foliacea oceanica* Heron-Allen & Earland, 1915

*Textularia lateralis* Lalicker, 1935**

*Textularia pseudorugosa* Lacroix, 1932**

*Textularia stricta* Cushman, 1911**

*Textularia* spp.

*Tretomphalus bulloides* (d'Orbigny, 1839)

*Tretomphalus concinnus* (Brady, 1884)

*Trichohyalus aguayoi* (Bermúdez, 1935)*

*Triloculina asymetrica* Said, 1949*

*Triloculina barnadi* Rasheed 1971

*Triloculina bicarinata* d'Orbigny, 1839

*Triloculina earlandi* Cushman, 1954

*Triloculina elongotricarinata* Debenay, 2013

*Triloculina fichteliana* d'Orbigny, 1839

*Triloculina* cf*. T. fichteliana* d'Orbigny, 1839

*Triloculina inflata* d'Orbigny, 1846**

*Triloculina laevigata* d'Orbigny, 1826**

*Triloculina linneana* d'Orbigny, 1839**

*Triloculina marioni* Schlumberger, 1893**

*Triloculina oblonga* (Montagu, 1803)

*Triloculina* cf. *T. oblonga* (Montagu, 1803)

*Triloculina planciana* d'Orbigny, 1839**

*Triloculina rotunda* Schlumberger, 1893

*Triloculina schreiberiana* d'Orbigny, 1839**

*Triloculina subgranulata* Cushman, 1918*

*Triloculina serrulata* McCulloch, 1977

*Triloculina* cf*. T. striatotrigonula* Parr, 1941

*Triloculina tricarinata* d´Orbigny, 1826*

*Triloculina* cf. *T. tricarinata* d'Orbigny, 1826*

*Triloculina trigonula* (Lamarck, 1804)*

*Triloculina triquetrella* Loeblich & Tappan, 1994

*Triloculina webbiana* d'Orbigny, 1839**

*Triloculina* spp.

*Triloculinella parisa* Loeblich & Tappan, 1994

*Triloculinella pseudooblonga* (Zheng, 1980)

*Trifarina bradyi* Cushman, 1923

*Trimosina orientalis* Cushman, 1933

*Trochammina inflata* (Montagu, 1808)*

*Ungulatella pacifica* Cushman, 1931**

*Uvigerina porrecta* Brady, 1879**

*Vagulina pauciloculata* (Brady, 1884)**

*Valvulineria candeiana* (d’Orbingy, 1839)**

*Vertebralina striata* d'Orbigny, 1826*

*Wiesnerella auriculata* (Egger, 1893)*
